# MTA2 is one of 14 Transcription factors predicting recurrence free survival in gastric cancer and promotes cancer progression by targeting MCM5

**DOI:** 10.7150/jca.77402

**Published:** 2023-01-01

**Authors:** Anshu Li, Yan Guo, Zhijie Yin, Xinghua Liu, Gengchen Xie

**Affiliations:** 1 Department of Gastrointestinal Surgery, Union Hospital, Tongji Medical College, Huazhong University of Science and Technology, Wuhan, 430022, China.; 2 Department of Chronic Noncommunicable Diseases Control and Prevention, Wuhan Center for Disease Control and Prevention, Wuhan, Hubei, China.

**Keywords:** gastric cancer, GEO, TCGA, MTA2, MCM5

## Abstract

Gastric cancer (GC) is a leading cause of cancer-related death worldwide. Transcription factors (TFs) are essential gene expression regulators, and play critical roles in cancer development. However, the biological actions and prognostic value of TFs in GC remain unclear. In this study, we identified a risk model based on a 14-TF signature to predict recurrence-free survival in patients with GC. We further analyzed the ability of 14-TF to predict recurrence-free survival time in GC and found that a higher expression level of metastasis-associated protein 2 (MTA2) was associated with shorter overall survival and disease-free survival time in GC. Through *in vitro* and *in vivo* experiments, we demonstrated that MTA2 significantly promotes GC growth and metastasis. Furthermore, we identified MTA2 binding to the promoter of minichromosome maintenance deficient 5 (MCM5), thereby promoting GC progression. Overall, these findings strongly support the prognostic potential of the 14-TFs signature and suggest that targeting MTA2 may be a promising strategy to treat GC.

## 1. Introduction

Gastric cancer (GC) is currently the fifth most common malignancy and third most common cause of cancer-related deaths globally 1, 2. Despite efforts to improve the survival of patients with GC, a favorable prognosis has not yet been obtained 3. Therefore, the identification of new GC prognostic factors and therapeutic targets is highly warranted.

Transcription factors (TFs) are major contributors to cancer progression and prognosis 4, 5. For instance, Edwards et al. revealed that ZEB1 serves as a TF that is prognostic and predictive in diffuse gliomas 6. Oktay et al. indicated that thyroid TF-1 plays a key role in lung adenocarcinoma prognosis 7. Tang et al. suggested that FOXO1 inhibits prostate cancer cell progression by suppressing E2F1-activated NPRL2 expression 8. Fan et al. reported that microRNA-301a-3p overexpression might contribute to cell invasion and proliferation by targeting runt-related TF3 in prostate cancer 9. Therefore, elucidating core transcriptional regulatory programs will provide a better understanding of molecular carcinogenesis. However, few studies have revealed the key roles of TFs in GC progression and prognosis.

In our study, we obtained gene expression data and clinical information for GC from the TCGA and Gene Expression Omnibus (GEO) databases, and the corresponding TFs and eligible patients were determined to investigate the utility of TFs markers for GC prognosis. We identified a 14-TF signature for predicting the recurrence-free survival (RFS) of patients with GC using bioinformatic integrated analysis. Moreover, we found that upregulation of metastasis-associated protein 2 (MTA2) was strongly associated with poor GC patient survival. Mechanistically, MTA2 promotes GC progression by targeting Mini Chromosome Maintenance 5 (MCM5). These findings strongly support the prognostic potential of the 14-TF signature and indicate that the MTA2/MCM5 axis represents a new therapeutic target for GC.

## 2. Materials and methods

### 2.1 Data source and processing

We searched the TCGA and GEO databases using the TCGAbiolinks package 10 and the GEOquery package 11 to obtain gene expression data and related clinical information for GC. In total, 24991 genes and 407 GC patients with intact clinical information in the TCGA database were included. Cases without prognostic data or non-TF genes were excluded from subsequent analysis. TFs were determined using the TRRUST database 12. Raw expression matrix counts were converted to transcripts per million (TPM). Genes with zero expression in over 20% of the samples were excluded. Finally, 721 TFs and 384 patients with GC were identified using univariate Cox regression analysis. Similarly, raw data of GSE26253 were preprocessed and normalized using the robust multichip averaging (RMA) 13 method using the affy packages 14 of R (v4.0.2). Ultimately, 432 patients in the GSE26253 dataset were included in the external validation set. The least absolute shrinkage and selection operator (LASSO) method was used to identify candidate TFs to predict RFS in patients with GC. The LASSO COX regression model was conducted using a publicly available R package for 1000 iterations.

### 2.2 Identification of prognostic TFs signature

The association between TFs expression and patient RFS was evaluated using univariate Cox regression analysis to select TFs relevant to patients' RFS. Then, the determined TFs were used to perform LASSO analysis to select candidate TFs that were reliably associated with RFS. Subsequently, a multivariate Cox regression analysis was performed based on the candidate TFs for the predictive TF signatures in the survival RFS evaluation of patients with GC.

A total of 384 patients were randomly assigned to the training (n = 269) and internal validation (n = 115) sets. The training cohort was used to identify the prognostic TF signature. The internal validation set, external validation set, and entire TCGA dataset were used to validate our results. The TF risk score formula was then established to determine the survival RFS risk for each patient using the coefficients from multivariate Cox regression analysis. Patients with GC in each set were stratified into high-risk or low-risk groups with the corresponding median risk score as the cutoff point. Survival differences between the high-risk and low-risk groups in each set were weighed using the Kaplan-Meier method and compared using the log-rank test. ROC analysis was conducted to assess the sensitivity and specificity of survival prediction based on the TF risk score. The greater the AUC value, the more superior the model for hazard prediction. Then, a stratified analysis was performed based on the clinical parameters in the whole set. All ROC and Kaplan-Meier curves were drawn using R (version 4.0.2).

### 2.3 Construction of nomogram

Univariate and multivariate Cox proportional hazard analyses were performed based on score and other clinicopathological factors. The factors with *P* ≤ 0.05 from multivariate Cox proportional hazard analysis were employed to establish a nomogram via the 'rms' R package. The prognostic value of the nomogram was assessed using the C-index, ROC, and calibration plots. The nomogram outcome is listed in the calibration curve and the 45‐degree line implied ideal performance.

### 2.4 Cell culture and Clinical specimens

The human normal GES-1 gastric mucosa epithelial cell line and GC cell lines (MNK45, SGC7901, AGS, and BGC823) were purchased from the Cell Bank of the Type Culture Collection of the Chinese Academy of Sciences (Shanghai, China). All cells were cultured in Roswell Park Memorial Institute (RPMI) medium 1640 (Gibco, Grand Island, NY, USA) supplemented with 10% fetal bovine serum (FBS), 1% penicillin, and streptomycin. The cells were then incubated at 37 °C in a 5% CO_2_ incubator.

Fresh GC tissue samples and matched adjacent non-tumor tissues were obtained from patients (n=48) who underwent surgery at the Department of Gastrointestinal Surgery, Union Hospital, Tongji Medical College, Huazhong University of Science and Technology. None of the patients had received preoperative chemotherapy or radiotherapy before surgery. This study was approved by the hospital's institutional review board, and written informed consent was obtained from each patient. All diagnoses were confirmed via histopathological examination.

### 2.5 Quantitative Reverse Transcription-Polymerase Chain Reaction (qRT-PCR)

For mRNA or miRNA expression level examination, total RNA from tissues or cells was isolated using the TRIzol reagent (TaKaRa Bio, Tokyo, Japan). cDNA was synthesized using the PrimeScript RT Reagent Kit (TaKaRa Bio, Shiga, Japan). The mRNA and miRNA expression levels were detected by qRT-PCR using the SYBR-Green Master mix (Takara). Glyceraldehyde-3-phosphate dehydrogenase (GAPDH) was used as endogenous control and the fold change was calculated using the relative quantification method (2^-ΔΔCt^). The related primer sequences are listed in Supplementary **Table S1**.

### 2.6 Cell Transfection

The cells were seeded in 6-well plates (2 × 10^4^ cells/well) and then transfected with the recombinant plasmid (Genechem Company, Shanghai, China) for 12 h. Transfection was performed using Lipofectamine 3000 (Invitrogen, Carlsbad, CA) following the manufacturer's instructions. Before further experiments, the efficacy of gene knockdown or overexpression was measured using qRT-PCR and western blot analysis.

### 2.7 Transwell

For the migration assay, MNK45 and BGC823 cells were transfected with a recombinant plasmid for 72 h and then added into the top chamber (Corning Costar, Rochester, NY) at a density of 1 × 10^5^ cells in a serum-free medium. For the invasive assay, the chamber was coated with Matrigel (BD Biosciences, Franklin Lakes, NJ, USA), and the subsequent steps were similar to those of the migration assay. Fresh culture media containing 20% FBS was added to the lower wells and incubated for 24 h at 37 °C with 5% CO_2_. The cells were fixed with 4% paraformaldehyde (PFA), stained with 0.5% crystal violet (Sigma-Aldrich, USA) for 20 min, and imaged using a microscope (Olympus IX71, Japan).

### 2.8 Animal Study

Five-week-old male BALB/c nude mice (Beijing Vital River Laboratory Animal Technology Co., Ltd.) were randomized into two groups (n=5 per group, total=10). Tumor cells (5×10^6^) were injected into right dorsal flanks of blindly randomized nude mice (n=5, 1×10^6^ per mice), respectively. Every 5 days, we measured the tumor's longitudinal diameter and latitudinal diameter using a caliper. Tumor volume was assessed using the formula: V = 0.5 × D × d^2^ (V, volume; D, longitudinal diameter; d, latitudinal diameter). The mice were sacrificed 25 days post-injection, and the tumor tissues were weighed. The animals were housed in cages under sterile, pathogen-free conditions. All *in vivo* protocols were approved by the Animal Research Committee of the Academic Medical Center at the Huazhong University of Science and Technology.

### 2.9 Western blot

Total protein was extracted from cells using radioimmunoprecipitation assay (RIPA) lysis buffer (Beyotime, Shanghai, China). The cell lysates were separated using 10% SDS polyacrylamide gel electrophoresis, and the separated proteins were transferred onto polyvinylidene difluoride (PVDF) membranes. Subsequently, the membranes were blocked with 5% non-fat milk in TBST for 1 h, followed by incubation of primary antibodies (GAPDH, 1:5000, ab8245, Abcam; MTA2, 1:1000, ab8106, abcam; MCM5, 1:1000, ab75975, Abcam) overnight at 4 °C. The membranes were then incubated with an HRP-conjugated secondary antibody for 1 h at room temperature. Specific bands were detected using Pierce ECL western blotting substrate (Thermo Fisher, 32109). Images were obtained and analyzed using the Image Lab software (version 3.0, National Institute of Health, Bethesda, MD, USA).

### 2.10 Chromatin immunoprecipitation (CHIP)

The ChIP assay was performed using the EZ ChIP™ Chromatin Immunoprecipitation Kit (Millipore, Billerica, Massachusetts, USA) according to the manufacturer's protocol. Briefly, MNK45 and BGC823 cells were cross-linked with 1% formaldehyde for 10 min at room temperature and quenched with 125 mM glycine. The target cells were collected, and the mixture was shredded to fragments of 200 bp via sonication. Immunoprecipitation was performed using the anti-MTA2 antibody (ab8106, Abcam) or control IgG. After purification, the enriched DNA-binding sites were analyzed using qRT-PCR.

### 2.11 Luciferase reporter assay

The possible targeting linkage between MTA2 and MCM5 was predicted by MiRWalk2.0 software and verified by luciferase reporter assay. Transfection of MNK45 cells was performed with the PMIR-REPORT luciferase vector with wild-type (WT)- MCM5 - 3'UTR or mutant (MUT)- MCM5 -3'UTR, MTA2 shRNA, or NC shRNA. After culturing for 48 h, the cells were harvested and luciferase signals were detected using a TECAN Infinite F500 platform with the Dual-Luciferase Reporter Assay System according to the manufacturer's protocols. The experiment was conducted at least thrice.

### 2.12 Cell proliferation assay

Cell proliferation was measured using Cell Counting Kit-8 (CCK-8; Dojindo, Japan) and colony formation assays. Briefly, the cells were seeded into 96-well plates at a density of 1×10^3^ cells/well. After cell culture for 1, 2, 3, 4, and 5 days, respectively, 10 μL CCK-8 solution was added to each well and incubated at 37 °C for 2 h. Absorbance at 450 nm was measured using a microplate reader (Thermo Scientific, Waltham, MA, USA). For the colony formation assay, 1.0×10^3^ treated cells were seeded in 6-well plates for approximately 14 days. These plates were washed twice with phosphate-buffered saline (PBS), fixed with 4% PFA, and stained with 0.1% crystal violet solution for further analysis.

### 2.13 Wound healing

Cells were seeded in 6-well plates at a high density and allowed to form cell monolayers overnight. The monolayers were scraped using a 200 μL sterile plastic tip to create a wound line and washed with PBS to remove the detached cells. Cells were cultured in a 1% FBS complete medium in a humidified 5% CO_2_ incubator at 37 °C. To visualize wound healing, images were taken with a phase-contrast microscope at 0 and 48 h post wounding. The wound closure percentage (original width-width after cell migration/original width) was calculated. Each assay was performed in triplicate.

### 2.14 Statistical analysis

Statistical analyses were performed using GraphPad Prism (version 7.0, GraphPad Software, Inc., La Jolla, CA, USA). Data are presented as mean ± standard deviation (SD). Significant differences were calculated using a two-tailed Student's t-test or one-way analysis of variance (one-way ANOVA). Experiments were independently repeated at least three times. Statistical significance was set at *P* < 0.05.

## 3. Results

### 3.1 Clinical characteristics of the study populations

**Figure 1:**
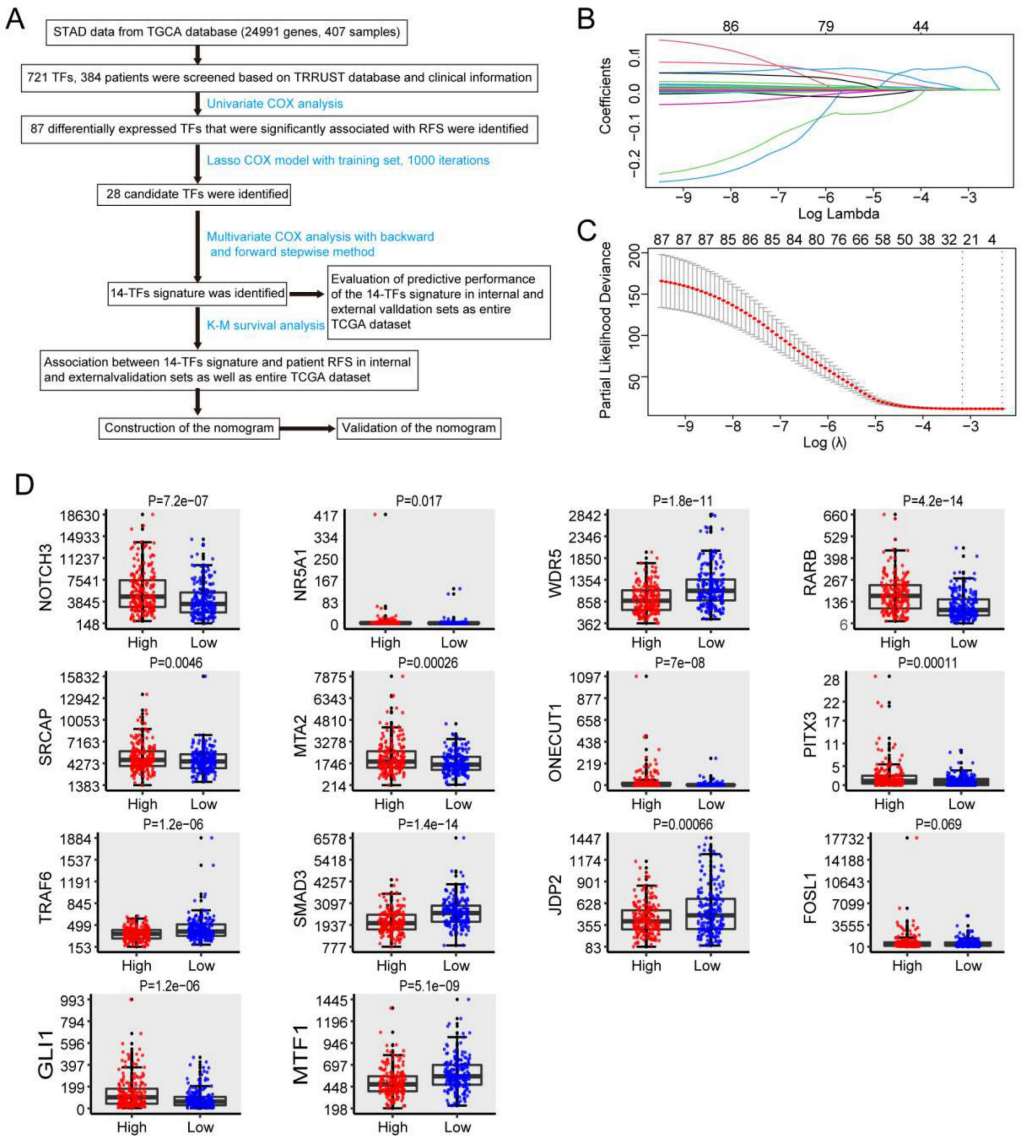
**Identification of TFs signature predicting RFS in patients with GC.** (**A**) Flow chart of the present study. (**B**) 10-fold cross-validation for tuning parameter selection in the LASSO model by minimum criteria (the 1-SE criteria). (**C**) LASSO coefficient profiles of the 87 TFs genes. A coefficient profile plot was conducted against log (lambda) sequence. Vertical line was drawn at the value selected with 10-fold cross-validation, in which optimal lambda led to 28 non-zero coefficients. (**D**) Boxplots of the 14 TFs expression values against risk group in the TCGA dataset. “High” and “Low” referred to the high-risk and low-risk clusters, respectively. The differences between the 2 clusters were evaluated by Mann-Whitney U test, and P values were observed in the graphs.

This study included 384 patients who were clinically and pathologically diagnosed with GC. Of these, 247 (64.32%) were male and 137 (35.68%) were female. The median age at diagnosis was 68 years (range, 35-90 years) and the median RFS was 383 days. All patients had an RFS rate of 10.4% over the 3 years. The pathologic stage was defined according to the American Joint Committee on Cancer (AJCC) Cancer Staging Manual. The stage of GC patients ranged from I to IV, with 56 (14.58%) patients in state I, 119 (30.99%) patients in stage II, 144 (37.5%) patients in stage III, 42 (10.94 %) in stage IV, and 23 (5.99%) patients in stage X (X: the stage cannot be identified). The histological type was separated into 3 groups, and 213 (55.49%), 170 (44.27%), and 1 (0.26%) patients were histologically classified as stomach adenocarcinoma and stomach-intestinal adenocarcinoma, respectively. Patients were separated into three groups according to the cancer status of the samples: 262 tumor-free (68.23%), 69 (17.97%), and 53 (13.80%). Additionally, the race list included Asian, Black, or African American, native Hawaiian or other Pacific Islander, White, and indeterminate. The white group was the most common 239 (62.24%). The complete list of clinicopathological characteristics of all included patients in the TCGA and GEO databases is detailed in **Table S2**. **Figure 1A** shows the flowchart of the study.

### 3.2 Identification of TFs significantly associated with RFS and establishment of prognostic signatures

Univariate Cox regression analysis and LASSO Cox regression analysis were conducted to identify the relationship between 721 TFs and RFS in patients with GC. As a result, 28 TFs were revealed to be significantly correlated with the RFS of GC patients after LASSO Cox regression analysis (**Figure 1B & 1C, Table S3**). Finally, 14 TFs (NOTCH3, NR5A1, WDR5, RARB, SRCAP, MTA2, ONECUT1, PITX3, TRAF6, SMAD3, JDP2, FOSL1, GLI1, MTF1) were found to be significantly related to RFS in GC patients by multivariate Cox analysis. Risk score = 6e-05*NOTCH3 + 0.00878*NR5A1 - 0.00124*WDR5 + 0.00233*RARB + 1e-04*SRCAP + 0.00025*MTA2 + 0.00217*ONECUT1 + 0.08996*PITX3 - 0.00186*TRAF6 - 0.00039*SMAD3 - 0.00233*JDP2 + 0.00012*FOSL1 + 0.00196*GLI1 - 0.00257*MTF1. The 14-TF signature was employed to predict the RFS of patients with GC. High TF expression of NOTCH3, NR5A1, RARB, SRCAP, MTA2, ONECUT1, PITX3, FOSL1, and GLI1 corresponded to a higher risk. Nevertheless, low TF expression of WDR5, TRAF6, MTF1, SMAD3, and JDP2 corresponded to higher risk (**Figure 1D**) (**Figure S1**).

### 3.3 Relationship between the 14-TF signature and patient RFS in internal validation dataset and external validation dataset as well as the whole dataset

**Figure 2:**
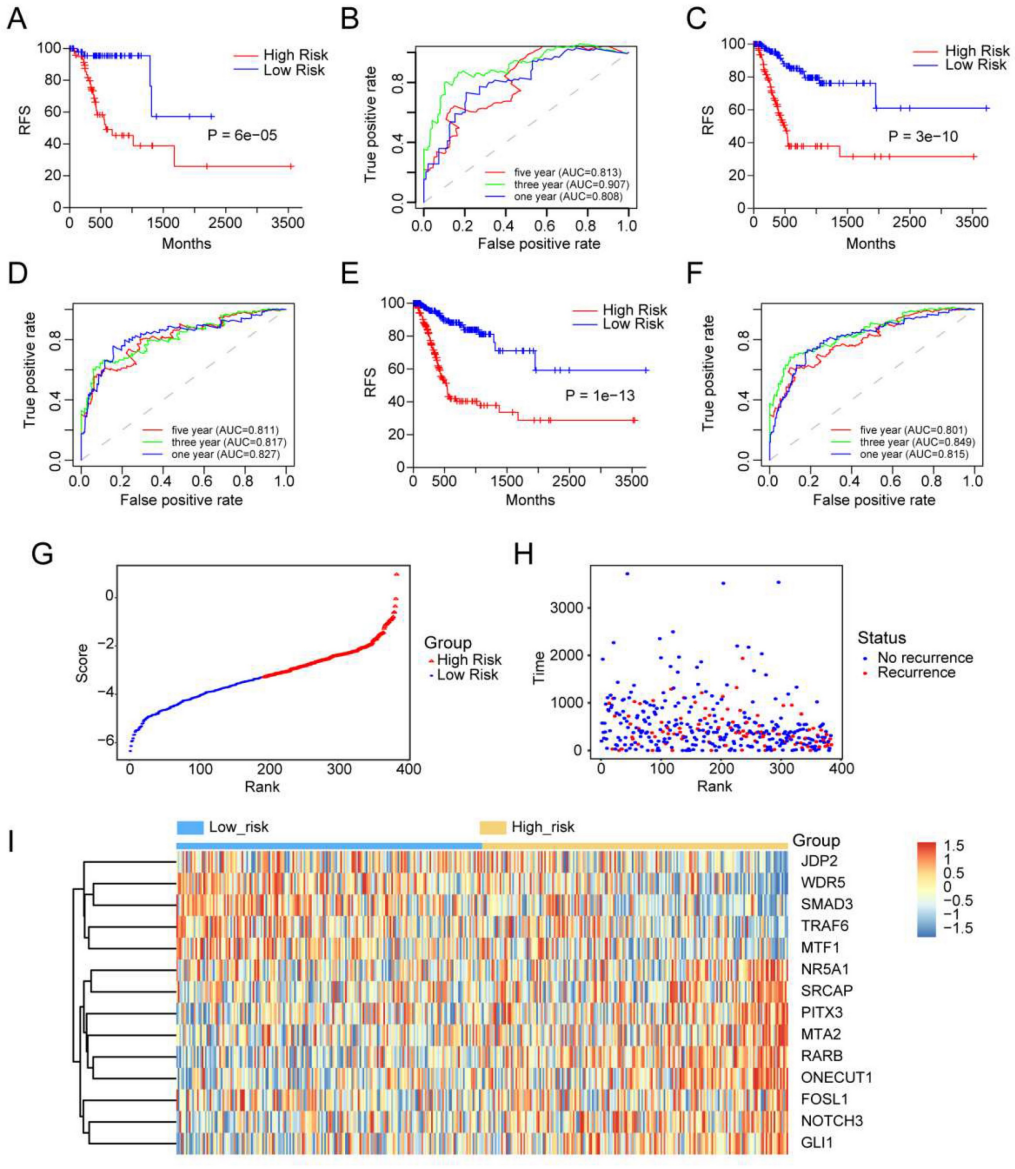
**Prognostic performance of the 14-TFs signature in GC. (A, C, E)** Kaplan-Meier analysis with two-sided log-rank test was performed to estimate the differences in RFS between the low-risk and high-risk patients. (**B, D, F**) 1-, 3-, 5-year ROC curves of the 14-TF signature were used to demonstrate the sensitivity and specificity in predicting the RFS of GC patients. (**G**) Risk score distribution against the rank of risk score. Median risk score served as the cut-off point. (**H**) Recurrence free survival status of GC patients. (**I**) Heatmap of 14 TFs expression profiles of GC patients.

Kaplan-Meier analysis was used to measure the difference in RFS between the two groups. RFS for high-score GC patients was shorter than that for low-score GC patients in the internal validation set (P=6e-05) (**Figure 2A**). A similar outcome was observed in the external validation dataset (p = 3e-10) (**Figure 2C**) and the entire dataset (p = 1e-13) (**Figure 2E**).

### 3.4 Evaluation of predictive performance of 14-TF signature by using ROC analysis

Time-dependent ROC curves were generated to assess the predictive power of the 14-TF signature. The AUC of the 14-TF signatures at 1, 3, and 5 years in the internal validation dataset were 0.813, 0.907, and 0.808, respectively (**Figure 2B**). A high predictive power was also observed in the external validation dataset (0.811, 0.817, and 0.827) (**Figure 2D**) and the entire dataset (0.801, 0.849, and 0.815) (**Figure 2F**). The results suggest that the 14‐TF signature is a stable RFS predictor in patients with GC.

Furthermore, patients were ranked according to their risk scores (**Figure 2G**), and a plot was drawn based on their survival status (**Figure 2H**). The outcome implied that the high-risk cohort had a higher mortality rate than the low-risk cohort. A heatmap of the 14 TFs grouped according to risk score is presented in **Figure 2I**, which confirmed our previous boxplot. Similar results were obtained for the GSE26253 dataset. Additionally, a subgroup analysis was performed using a few clinicopathological factors, including age, sex, stage, histologic type, anatomic site, and metastasis status. The results demonstrated a good predictive power of the 14-TF in most subgroups (**Figure S2-S7**).

### 3.5 Nomogram development

**Figure 3:**
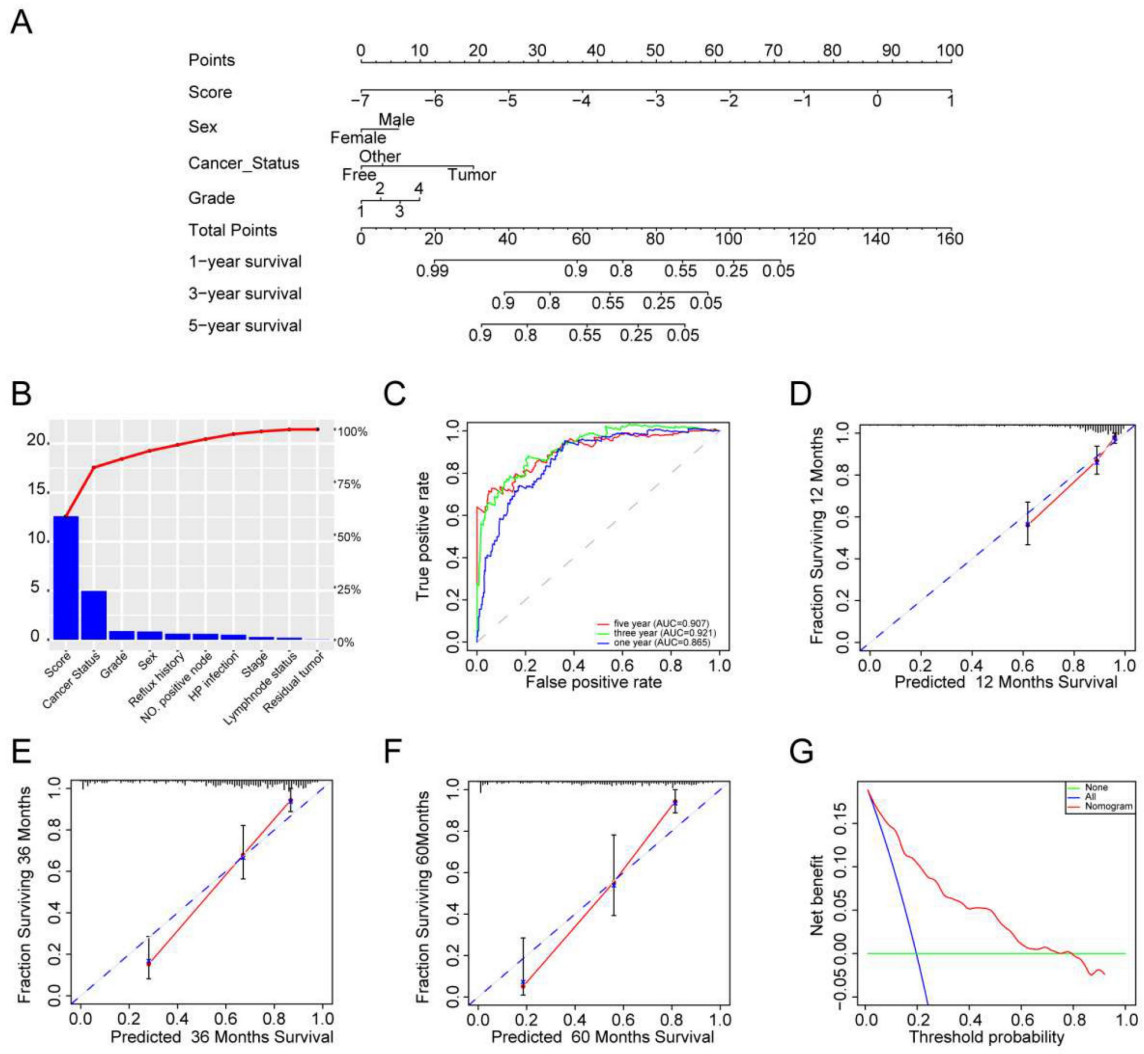
** Clinical applications of the 14 TFs signature.** (**A**) The nomogram was developed in the entire TCGA cohort, with the TFs risk score, sex, cancer status and tumor grade. (**B**) The higher the bar chart, the greater the importance. (**C**) 1-, 3-, 5-year ROC curves for the TFs-associated nomogram. (**D, E, F**) represent the 1-, 3-, 5-year nomogram calibration curves, respectively. The closer the dotted line fit is to the ideal line, the better the predictive accuracy of the nomogram is. (**G**) The DCA for the nomogram. The net benefit was plotted versus the threshold probability. The red line represented the nomogram. The blue line represented the treat-all and the green line represented the treat-none.

We performed univariate and multivariate Cox models via TF-related risk score and a few other clinicopathological factors to determine the independence of the 14-TF signature as a prognostic predictor in GC patients. Hazard ratios (HRs) demonstrated that the 14-TF signature was significantly correlated with the RFS of GC patients (*P* <0.001, HR 2.72, 95% CI 2.26-3.27) by Cox regression analysis (**Table S4**), implying that the 14-TF signature functioned as an independent prognostic predictor. Then we used the 14-TF signature as a single factor to perform univariate regression analysis with other clinical information. Factors with P value less than 0.05 were used for multivariate regression analysis. Finally, a nomogram (**Figure 3A**) combining the TFs risk score with other clinical factors (*P* < 0.2 in multivariate Cox analysis, Risk score, Sex, Cancer status, Tumor grade) were constructed and C-index (0.788, 95% CI: 0.741-0.835), AUC (0.865, 0.921, 0.907) (**Figure 3B & 3C**), and calibration plot presented a good performance (**Figure 3D-3F**), which yield higher predictive power than other models (0.776 for Shi et al., 0.83 for Lee et al., 0.718 for Jeong et al.) 15-17. DCA showed that the nomogram created a more crucial value of clinical utilization as an RFS predictor in GC patients than that in the treat all or treat none groups. Net benefits were available for GC patients with 3-year recurrence risks (**Figure 3G**). We conclude that our nomogram has great value and may have the potential for clinical applications.

### 3.6 MTA2 is upregulated in GC cells and tissues

**Figure 4:**
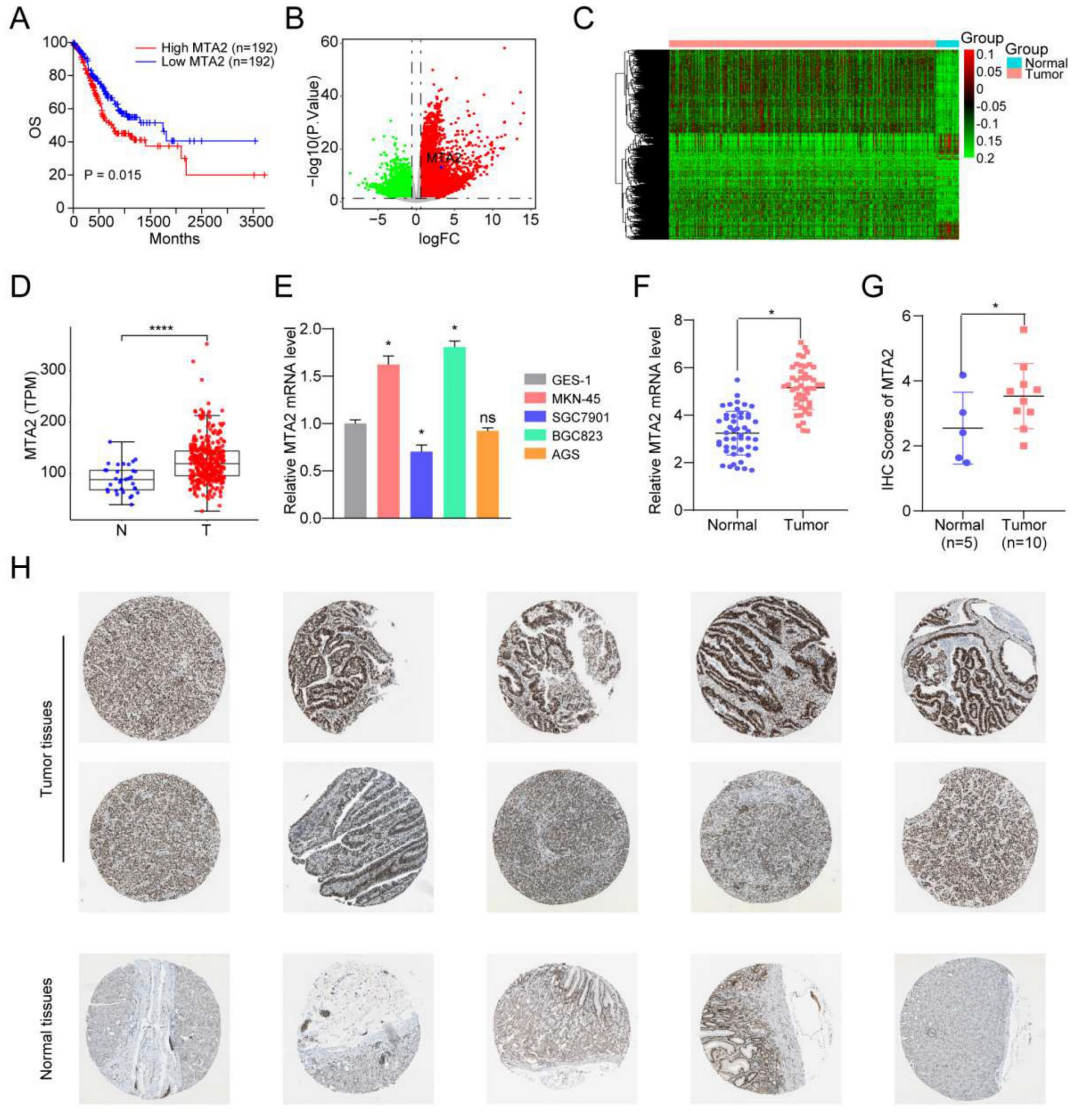
**The clinical-pathological feature of MTA2 in GC.** (**A**) The overall survival of MTA2 in GC analyzed based on TCGA-STAD RNA-seq data, P values as indicated. (**B**) The volcano plot of differentially expressed genes in TCGA-STAD datasets, read and green dots represents up-regulated and down-regulated genes, respectively; The blue dot represents MTA2. (**C**) The heatmap of differentially expressed genes in TCGA-STAD datasets. (**D**) The expression value the MTA2 in TCGA-STAD para-tumor group and tumor group. (**E**) The MTA2 genes were assessed by qRT-PCR assay in GES-1, MNK45, SGC7901, AGS and BGC823 cells. Data are presented as mean ± S.D. for at least three independent experiments. *P < 0.05. ns = not significant (**F**) The mRNA expression level of MTA2 analyzed by the collected GC samples, n.s., not significant. (**G, H**) The protein levels of MTA2 from the tissue microarray (n = 5 normal tissues, n = 10 GC tumor tissues) were determined by IHC analysis, Data are presented as mean ± S.D. *P < 0.05.

Next, TCGA-STAD RNA-seq data were used to analyze the ability of individual TFs of the signature to predict overall survival (OS) in GC. Intriguingly, the results showed that only MTA2 upregulation was correlated with shortened OS in patients with GC (**Figures 4 A**). MTA2 was upregulated in TCGA GC tissues (**Figures 4 B-4D**) and in our collected GC tissues (**Figure 4 F**). Compared to GES-1 cells, the MTA2 mRNA expression level was significantly increased in MNK45 and BGC823 cells. Additionally, MTA2 mRNA and protein levels (from the HPA database) were significantly increased in GC tissues relative to non-tumor tissues (**Figures 4G-H**), suggesting that MTA2 may play a vital role in GC progression.

### 3.7 MTA2 promotes proliferation, invasion and migration of GC cells

**Figure 5:**
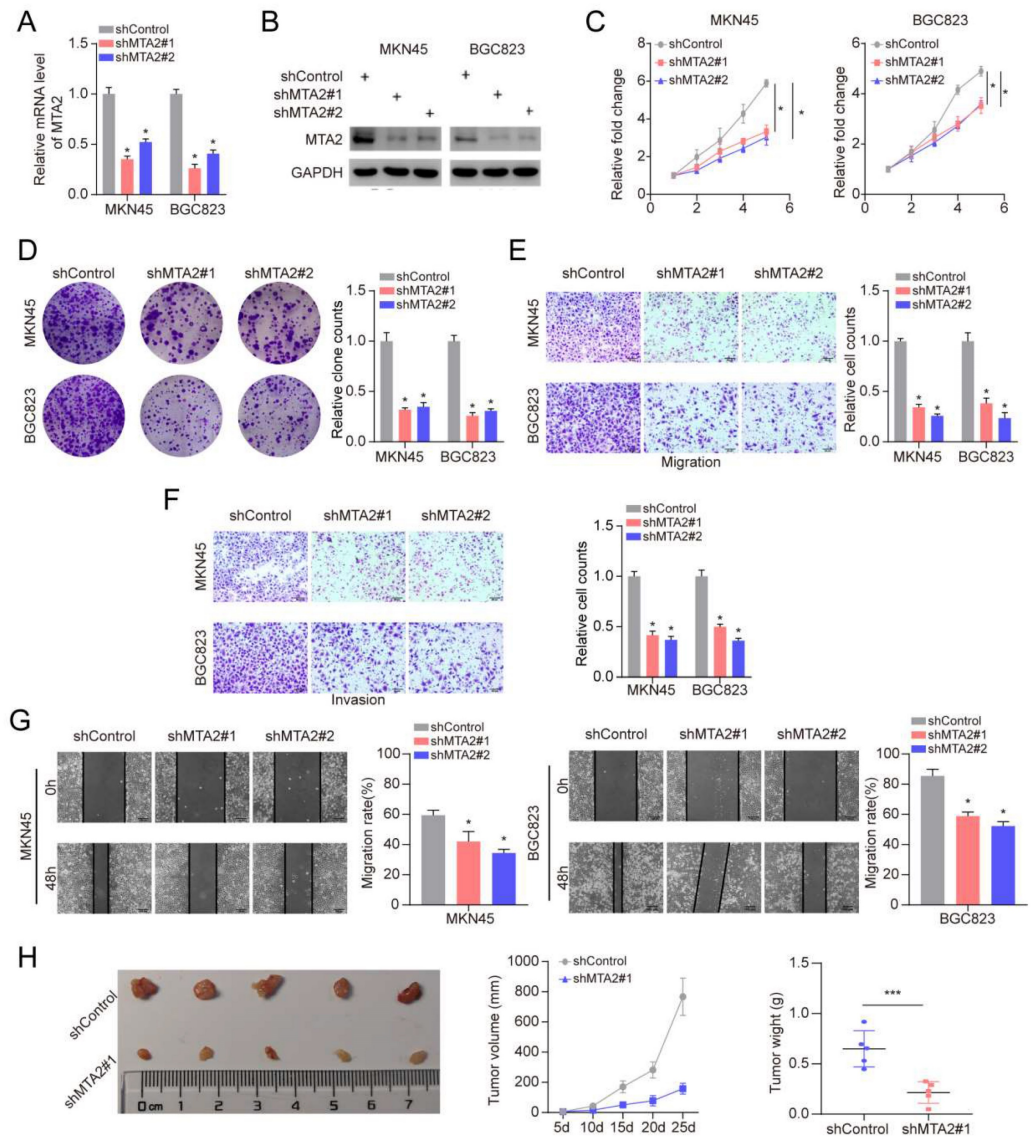
** Effects of MTA2 on GC cell proliferation, invasion and migration.** (**A, B**) RT-qPCR (A) and Western blot analysis (B) of MTA2 expression in MKN45 and BGC823 cells treated with indicated shRNAs. Data are presented as mean ± S.D. for at least three independent experiments. *P < 0.05. (**C, D**) The proliferative ability of MKN45 and BGC823 cells was investigated via cell viability and colony formation. Data are presented as mean ± S.D. for at least three independent experiments. *P < 0.05. (**E, F**) Transwell assay was exploited to explore the invasive and migratory ability with indicated shMTA2 in MKN45 and BGC823 cells. Data are presented as mean ± S.D. for at least three independent experiments. *P < 0.05. (**G**) Mobility was evaluated by wound-healing assay. Scale bars = 100μm. Data are presented as mean ± S.D. for at least three independent experiments. *P < 0.05. (**H**) Images of tumors after removal from the mice. MKN45 cells were transfected with shMTA2 or shControl and then injected subcutaneously into nude mice (n=5), respectively. Tumor growth curve. Tumor volumes were measured every five days after injection of tumor cells. Tumor weight when tumors were harvested. Data are presented as mean ± S.D. ***P < 0.001.

To further elucidate the role of MTA2 in GC, we knocked down MTA2 expression in MNK45 and BGC823 cells using two short hairpin RNAs (shRNAs) (**Figures 5A and 5B**). MTA2 downregulation inhibited cell proliferation and colony formation in MNK45 and BGC823 cells (**Figures 5C and 5D**). Additionally, the effects of MTA2 on the migratory and invasive abilities of MNK45 and BGC823 cells were assessed using wound healing and transwell assays. The results showed that MTA2 downregulation significantly blocked the migration and invasion of MNK45 and BGC823 cells (**Figure 5E-G**). Additionally, we investigated the role of MTA2 in promoting tumor growth *in vivo* by constructing a subcutaneous xenograft model. The results showed that the downregulation of MTA2 significantly reduced tumor volume and weight (**Figures 5H**). These results demonstrated that MTA2 plays a crucial role in the progression of GC cells.

### 3.8 MTA2 regulates MCM5 expression by binding to the promoter region of MCM5

**Figure 6:**
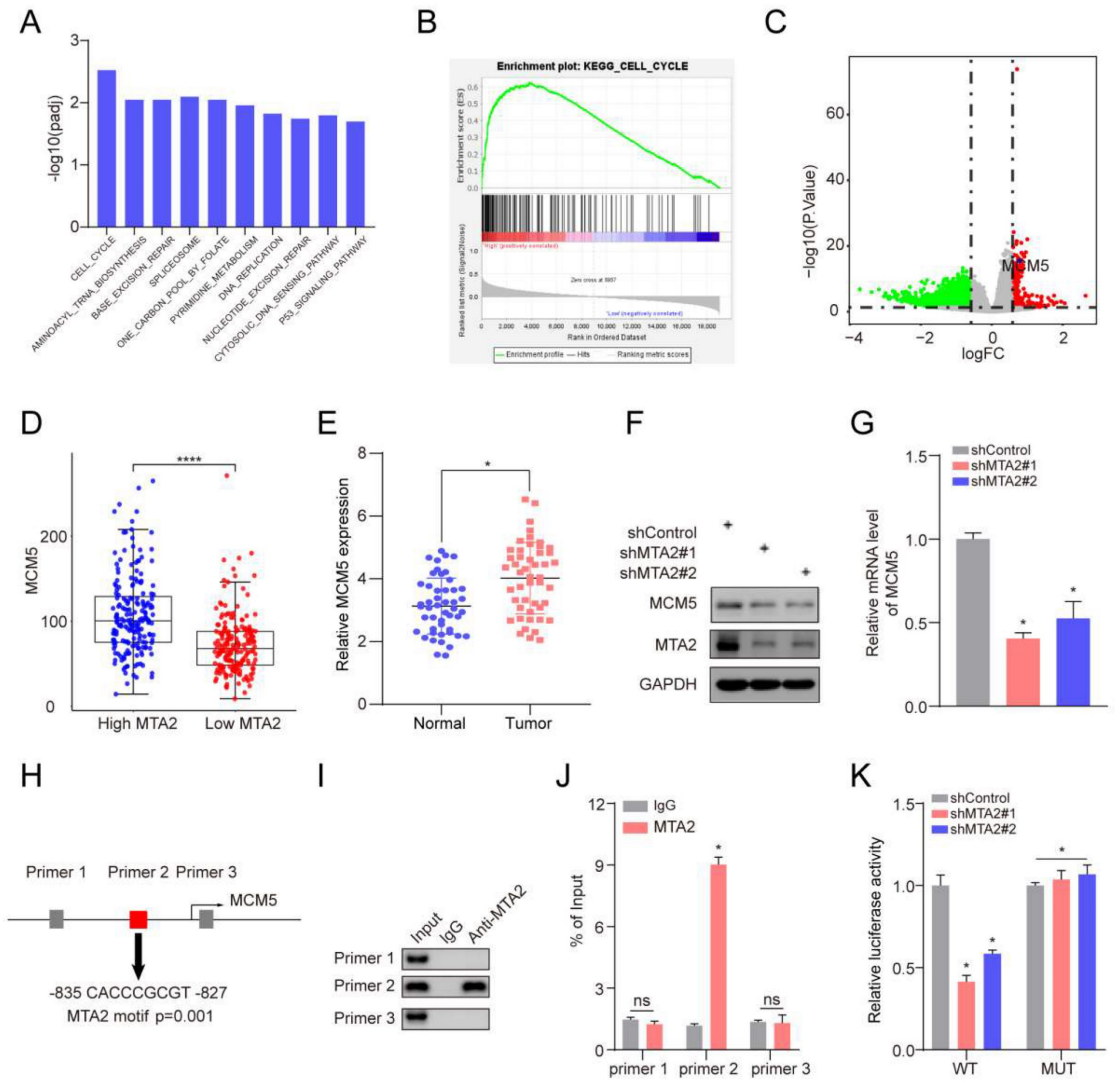
** MCM5 is the target gene of MTA2 in GC.** (**A**) Top 10 enriched pathways based on gene sets enrichment analysis (KEGG). (**B**) The cell cycle is the most significant enriched pathway. (**C**) The volcano plot of differentially expressed genes between MTA2 high group and low group, read and green dots represents up-regulated and down-regulated genes, respectively; The blue dot represent MCM5. (**D**) Comparison of the expression level of MCM5 between MTA2 high expression group and low expression group. (**E**) Comparison of the expression level of MCM5 between our collected tumor tissue and para-tumor tissue. (**F**) 72 h post-infection, MKN45 cells were infected with shControl or shMTA2 were harvested for Western blotting analysis. (**G**) The knockdown efficiencies of MTA2 were verified by qRT-PCR analysis. Data are presented as mean ± S.D. for at least three independent experiments. *P < 0.05. (**H**) The promoter region of MCM5 was searched from The Eukaryotic Promoter Database exhibit that there was three potential MTA2 binding sites in the promoter region. (**I**) ChIP experiments in MKN45 cells with the antibodies against MTA2 or with isotypic IgG as negative controls. (**J**) The ChIP-qPCR analysis of MKN45 cells. Data are presented as mean ± S.D. for at least three independent experiments. *P < 0.05. (**K**) The luciferase reporter assay in MKN45 cells co-transfected with the wild-type (WT) or mutant (Mut) MCM5 promoter luciferase reporter and the vector or MTA2 constructs. Schematic of the sequence of the putative consensus MTA2-binding element in the human MCM5 promoter region and the substitution mutations introduced into this binding element sequence are shown. The luciferase reporter activity results were depicted as a bar graph with mean ± S.D. n = 3. *P < 0.05.

**Figure 7:**
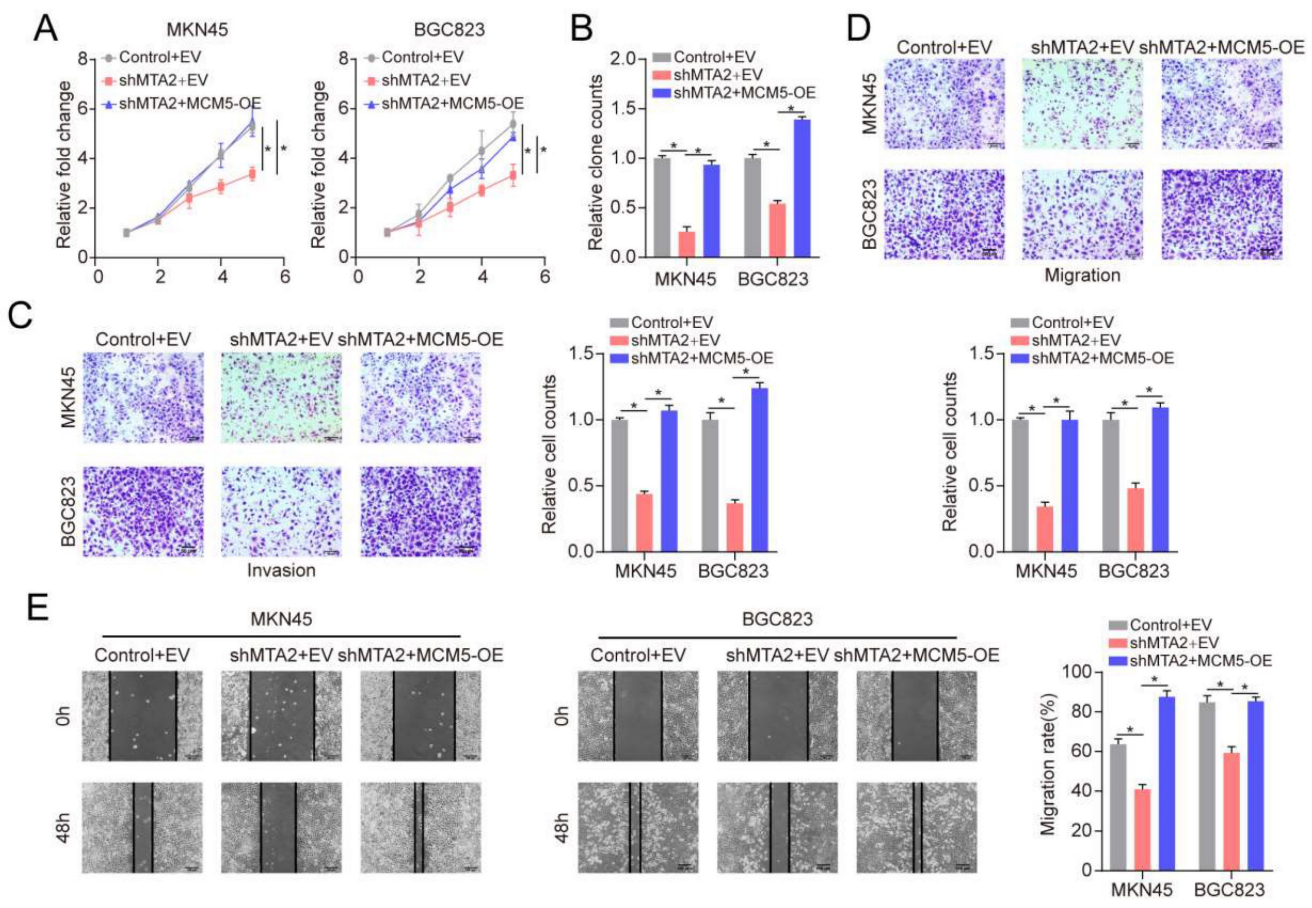
** MTA2 regulates the progression of GC cell via MCM5.** (**A, B**) CCK-8 and colony formation assays were performed to examine cell proliferation in MKN45 or BGC823 cells infected with shControl, shMTA2, or shMTA2 plus MCM5-overexpression (MCM5-OE), respectively, at the indicated time points. Data are presented as mean ± S.D. for at least three independent experiments. *P < 0.05. (**C, D**) MKN45 or BGC823 cells were infected with shControl, shMTA2, or shMTA2 plus MCM5-OE, respectively. (**C**) Cell migration or (**D**) invasion was determined using a transwell migration or invasion assay, respectively. Data are presented as mean ± S.D. for at least three independent experiments. *P < 0.05. (**E**) The migration of MKN45 or BGC823 cells in different treatment groups was tested by the wound healing assay. Scale bars = 100μm. Data are presented as mean ± S.D. for at least three independent experiments. *P < 0.05.

To explore how MTA2 plays the tumor-promoting role, we divided TCGA-STAD tumor samples into two groups according to the median expression value of MTA2 for GSEA analysis. KEGG enrichment analysis revealed that high MTA2 expression was positively correlated with the cell cycle pathway (**Figure 6A and 6B**). Interestingly, as a cell cycle pathway member, MCM5 was highly expressed in GC tumor tissue relative to non-tumor tissue (**Figures 6C and 6E**) and positively correlated with MTA2 (**Figure 6D**). MTA2 expression downregulation significantly decreased MCM5 protein and mRNA expression in MNK45 cells (**Figures 6F and G**). MCM5 promoter analysis using the eukaryotic promoter database (https://epd.epfl.ch//index.php) revealed three MTA2 binding sites (**Figure 6H**). Next, we designed primers for these three binding sites and found that only the Primer 2 region (-835 CACCCGCGT -827) could be pulled down by the MTA2 antibody (**Figure 6 I and J**). As shown in **Figure 6 K**, luciferase activity in the WT MCM5 promoter was decreased in MNK45 cells with downregulated MTA2 expression. However, no alteration was found in the MCM5 binding site at -835 to -827 bp upstream of the pre-MTA2 promoter region. These results indicate that MTA2 directly regulates MCM5 expression and activity in GC cells. Moreover, MTA2 expression downregulation suppressed the proliferation, invasion, and migration of GC cells (*P* < 0.05), and this effect could be rescued by high MCM5 expression (**Figure 7A-E**). Collectively, these data showed that MTA2 could exert its function in GC cells by regulating MCM5 expression.

## 4. Discussion

GC remains a severe public health challenge worldwide. Currently, prognostic models for GC are mainly based on the Union for International Cancer Control (UICC) tumor-node-metastasis (TNM) staging system. However, the results for patients with a similar TNM stage yield great differences owing to inherent heterogeneity 18-21. The identification of novel prognostic predictors and the establishment of more valuable therapeutic targets are urgently required.

In this study, we identified a combination of 14 TFs (NOTCH3, NR5A1, WDR5, RARB, SRCAP, SMAD3, ONECUT1, PITX3, TRAF6, MTA2, JDP2, FOSL1, GLI1, MTF1) and effectively predicted RFS in GC patients using the univariate Cox proportional hazard analysis, the LASSO Cox regression analysis, and multivariate Cox proportional hazard analysis. Various experiments have indicated that these 14 TFs are important in cancer development. For instance, Ganguly et al. reported that Notch3 promoted prostate cancer-induced bone lesion development via MMP-3 22. Impaired steroidogenic factor 1 (NR5A1) activity plays a significant role in mutant Y1 mouse adrenocortical tumor cells 23. The expression of WD repeat domain 5 (WDR5) is relevant to papillary thyroid carcinoma progression and reduced prognosis 24. Methylation of L1RE1, RARB, and RASSF1 serve as potential biomarkers for the differential diagnosis of lung cancer 25. The chromatin remodeling factor SRCAP regulates prostate-specific antigen expression and cellular proliferation in prostate cancer cells 26. MicroRNA-17 serves as an oncogene by downregulating smad3 expression in hepatocellular carcinoma 27. ONECUT1 expression loss plays a key role in human pancreatic cancer cells 28. PITX3 DNA methylation functions as an independent biomarker for predicting overall survival of patients with head and neck squamous cell carcinoma 29. TRAF6 enhances colorectal cancer progression and growth by nuclear shuttle regulation of the NF-κB/c-Jun signaling pathway 30. miR-1236-3p suppresses invasion and metastasis in gastric cancer by targeting MTA2 31. ATF3 and JDP2 deficiency in cancer-related fibroblasts enhances tumor growth via SDF-1 transcription 32. FOSL1 promotes the growth and metastasis of human prostate cancer cells via epithelial mesenchymal transition pathway 33. DZIP1 enhances proliferation, migration, and invasion of oral squamous carcinoma via the GLI1/3 pathway 34. Tumors expressing the mtfl-mrfp1-wttk fusion reporter play a key role in the construction and validation of improved triple fusion reporter gene vectors for the molecular imaging of living subjects 35. Therefore, these 14 TFs may play important roles in GC development and progression. Intriguingly, we found that MTA2 upregulation correlated with shortened RFS and OS in patients with GC.

MTA2 is a member of the metastasis-associated gene family is reportedly closely associated with tumor progression 36-38. For example, Zhu et al. found that the invasion and proliferation abilities of pancreatic carcinoma cells were reduced after MTA2 gene knockdown 39. In this study, we showed that the MTA2 gene and protein were overexpressed in GC cells and tissues. MTA2 expression downregulation significantly inhibited proliferation, migration, and invasion of GC cells. A xenograft tumor model study also showed that MTA2 knockdown inhibits tumor growth *in vivo*. These results indicate that MTA2 participates in GC progression and may be a potential therapeutic target for GC.

MTA2 is a core subunit of the nucleosome remodeling and deacetylating (NuRD) complex and is implicated in gene transcription repression 40. Previous studies have found that MTA2 regulates MMP12 expression and is involved in cervical cancer metastasis 41. MTA2 promotes proliferation, migration, and invasion of pancreatic ductal adenocarcinoma cells via transcriptional repression of phosphatase and tensin homolog (PTEN) 42. Indeed, it has been reported that MTA2 might be a predictor of aggressive phenotypes and a possible target molecule for anticancer drug design in GC 31, 43. However, the precise function and regulatory mechanism of MTA2 in GC have not yet been well characterized. Our findings revealed that MTA2 and MCM5 are highly expressed in GC tissues. Moreover, ChIP-seq and luciferase reporter analyses revealed that MTA2 binds to the MCM5 promoter (-835--827 bp) and that MCM5 was transcriptionally promoted by MTA2. MTA2 knockdown attenuates the proliferation, migration, and invasion of GC cells, both of which are attributed to MCM5 expression inhibition. Furthermore, MCM5 is frequently upregulated in various human cancers. Higher MCM5 expression is associated with a high tumorigenesis rate and a poor prognosis 44, 45. Therefore, transcriptional promotion of MCM5 by MTA2 may be a key mechanism in regulating GC tumor growth.

In summary, we established a 14-TF signature that accurately and reliably predicted the prognosis of patients with GC. Additionally, we systemically investigated the role of MTA2 in GC and found that MTA2 promotes GC cell proliferation, migration, and invasion via the transcriptional repression of MCM5. Our findings indicate that MTA2 is a potential therapeutic target for GC.

## Supplementary Material

Supplementary figures and tables.
