# Supplementary material for: MTA2 is one of 14 Transcription factors predicting recurrence free survival in gastric cancer and promotes cancer progression by targeting MCM5
Source: J Cancer. 2023 Jan 1;14(2):262–74. doi: 10.7150/jca.77402 (PMC9891871; doi:10.7150/jca.77402)
Supplement: Supplementary file 1 — Supplementary figures and tables. [file jcav14p0262s1.pdf]

Figure S1. Boxplots of 14 TFs expression values against risk group in the GSE26253.

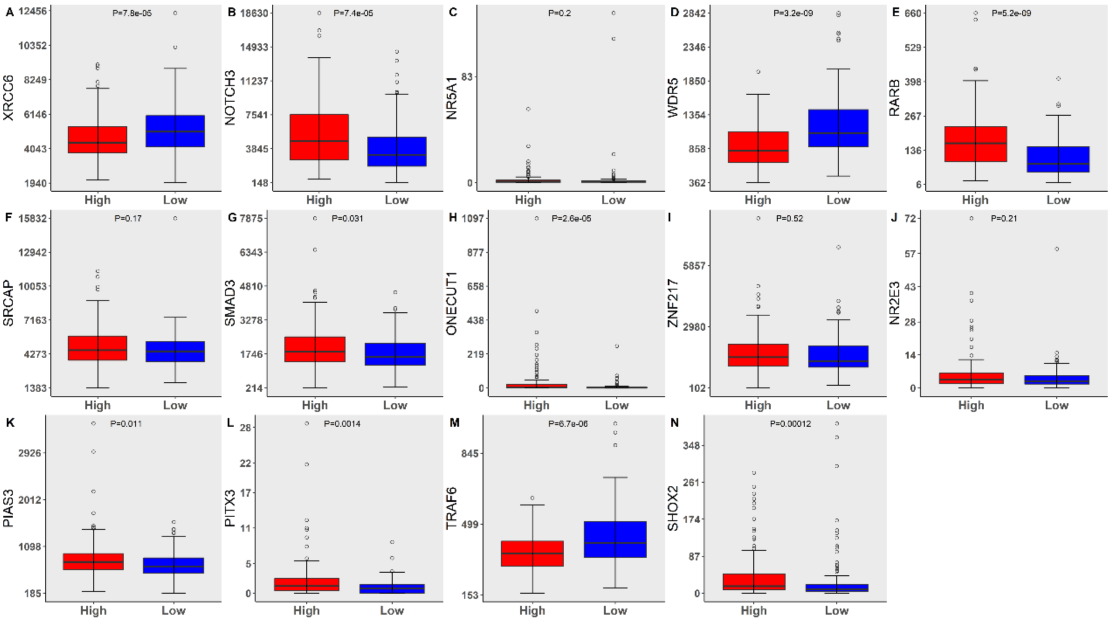

Figure S2. Kaplan-Meier and ROC analysis of patients with GC in sub-groups according to age, respectively. A), B) Age less 65 years sub-group. C), D) Age over 65 years sub-group.

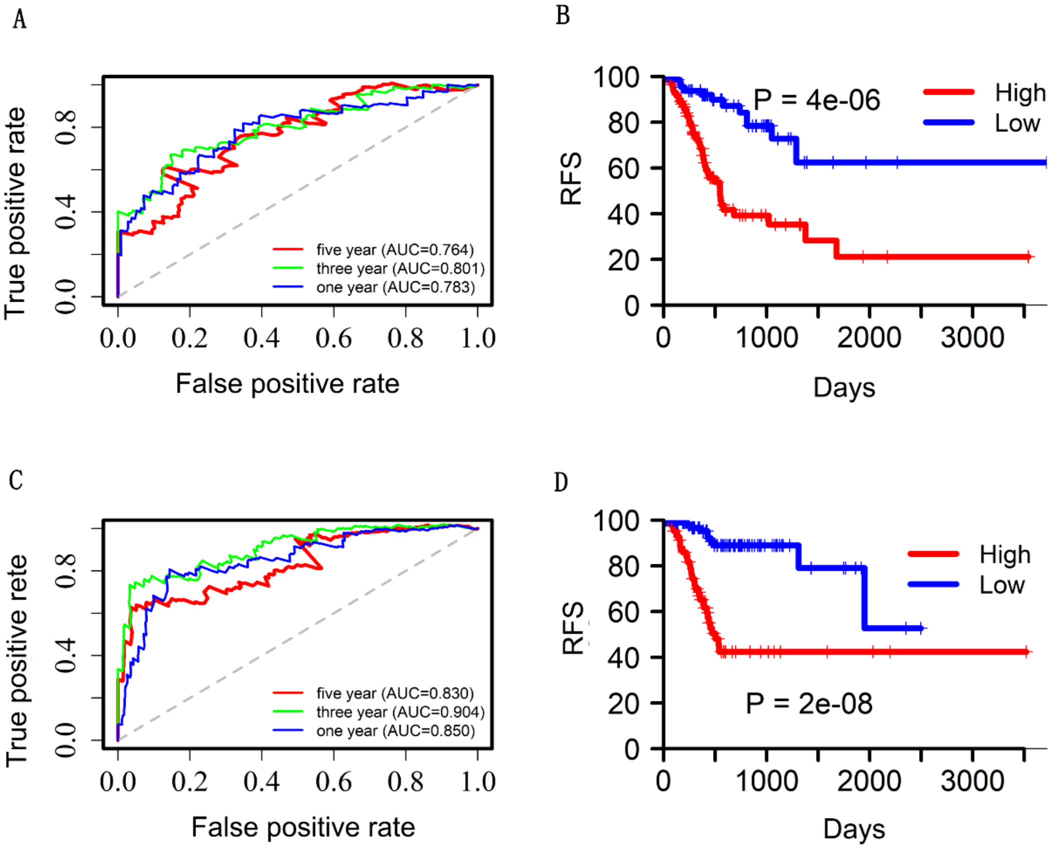

Figure S3. Kaplan-Meier and ROC analysis of patients with GC in sub-groups according to gender, respectively. A), B) Female sub-group. C), D) Male sub-group.

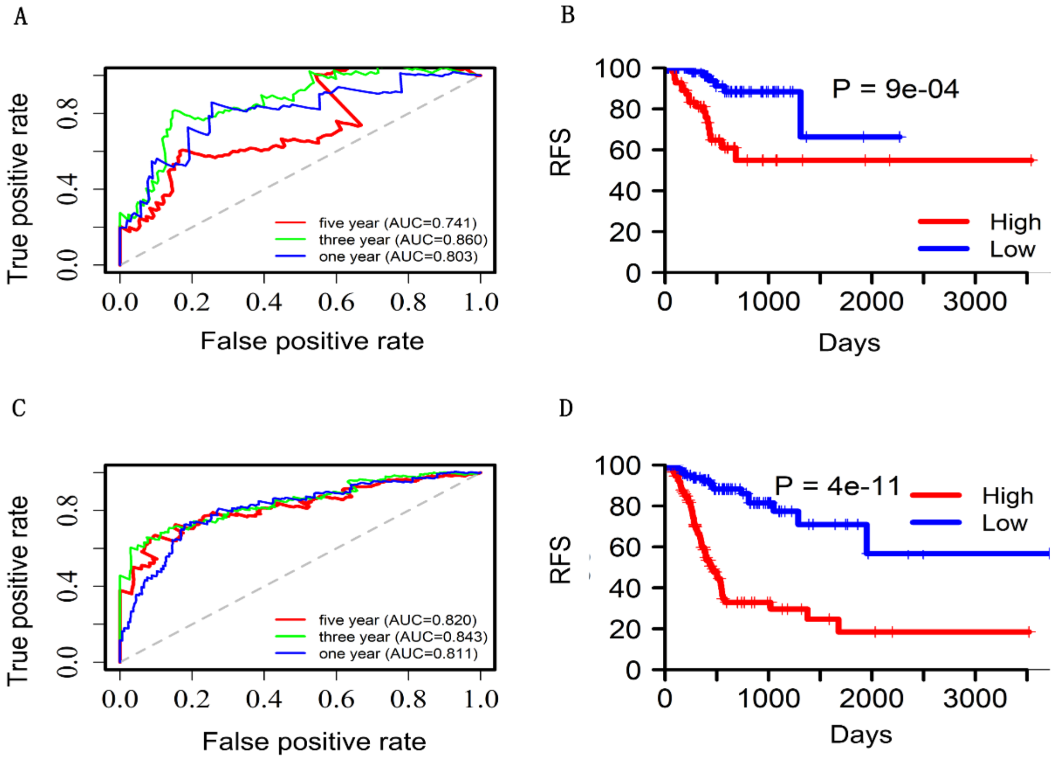

Figure S4. Kaplan-Meier and ROC analysis of patients with GC in sub-groups according to stage, respectively. A), B) Early stage sub-group. C), D) Advanced stage sub-group.

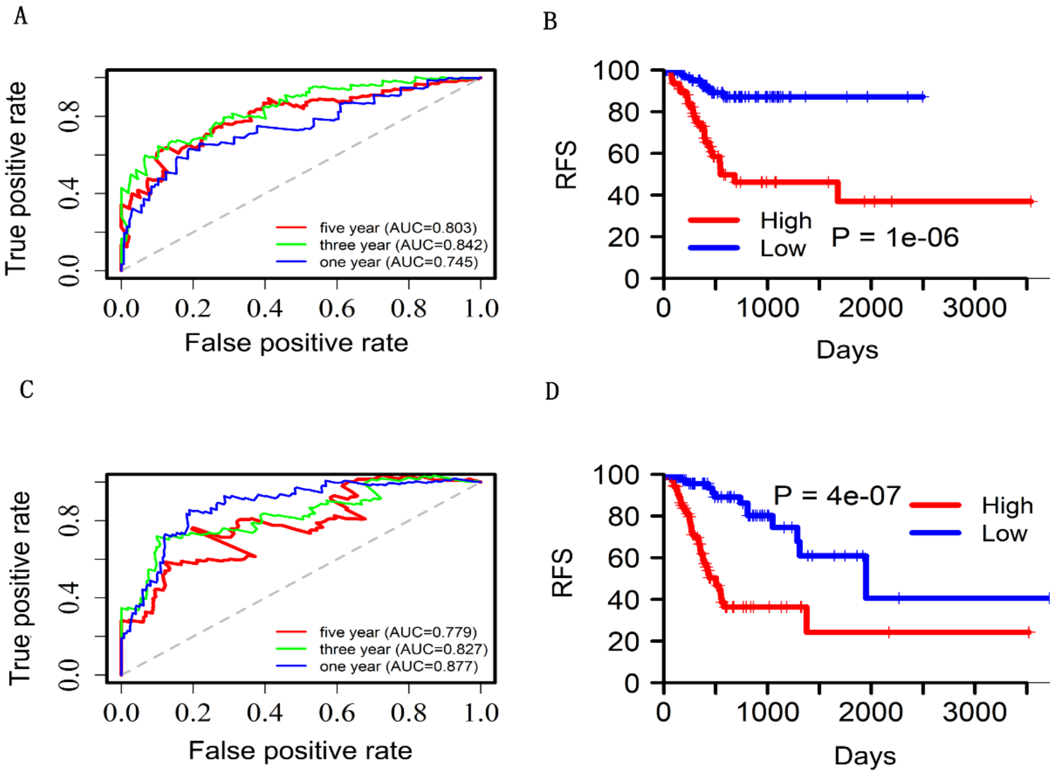

Figure S5. Kaplan-Meier and ROC analysis of patients with GC in sub-groups according to histologic type, respectively. A), B) Stomach type sub-group. C), D) Intestinal type sub-group.

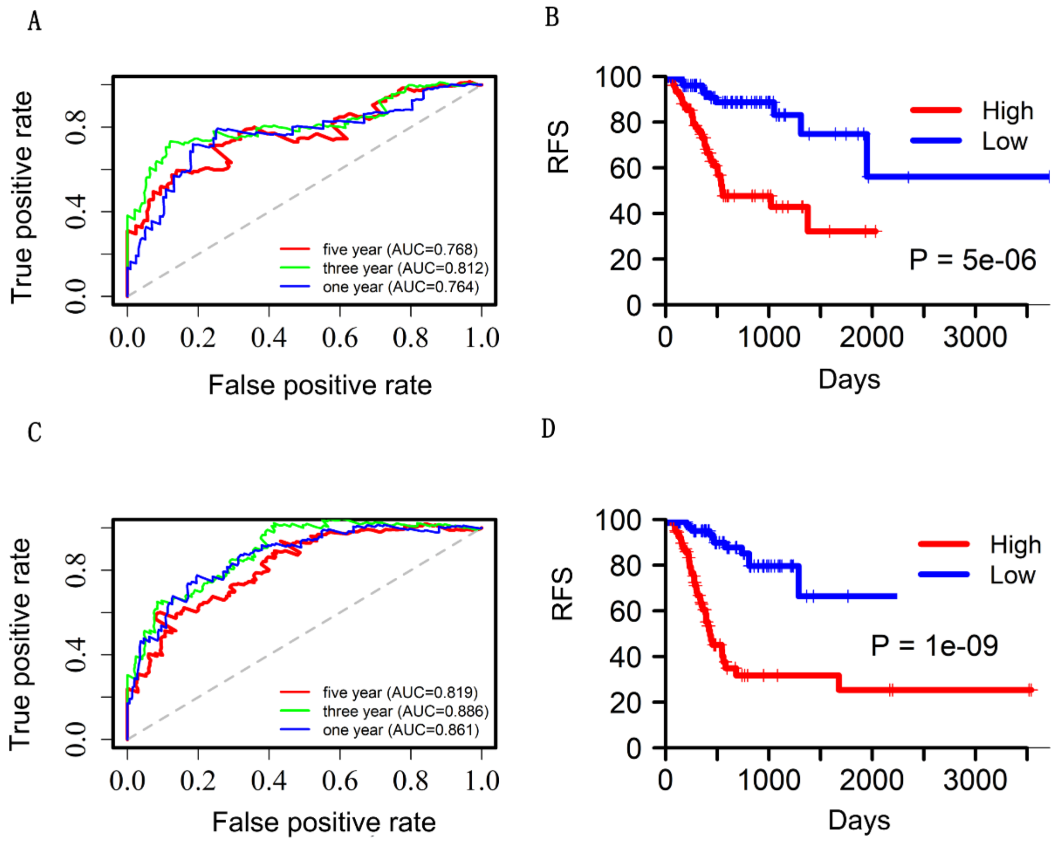

Figure S6. Kaplan-Meier and ROC analysis of patients with GC in sub-groups according to anatomic sites, respectively. A), B) Antrum sub-group. C), D) Cardia sub-group. E), F) Fundus sub-group. C), D) Junction sub-group.

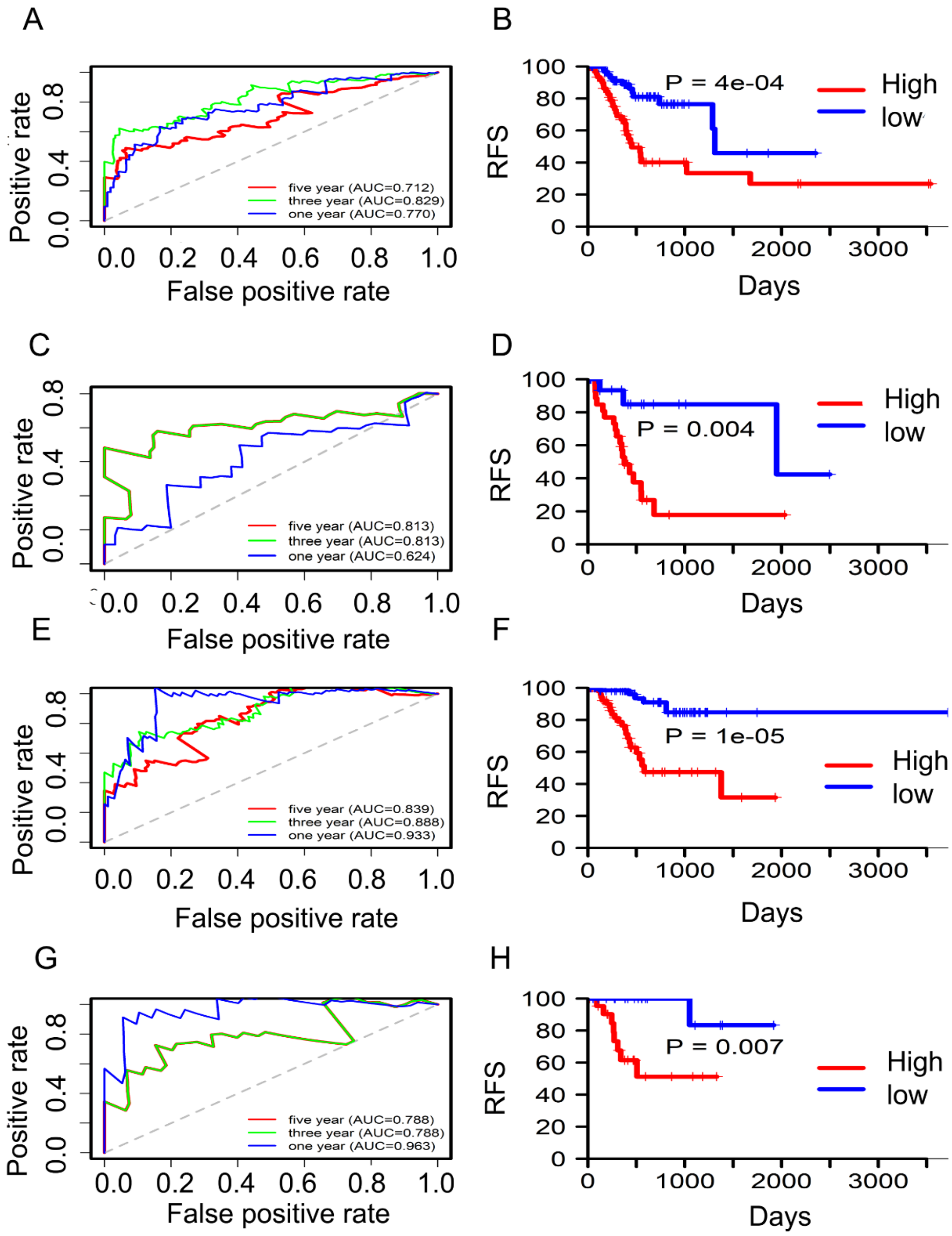

Figure S7. Kaplan-Meier and ROC analysis of patients with GC in sub-groups according to metastasis status, respectively. A), B) No metastasis sub-group. C), D) Metastasis sub-group.

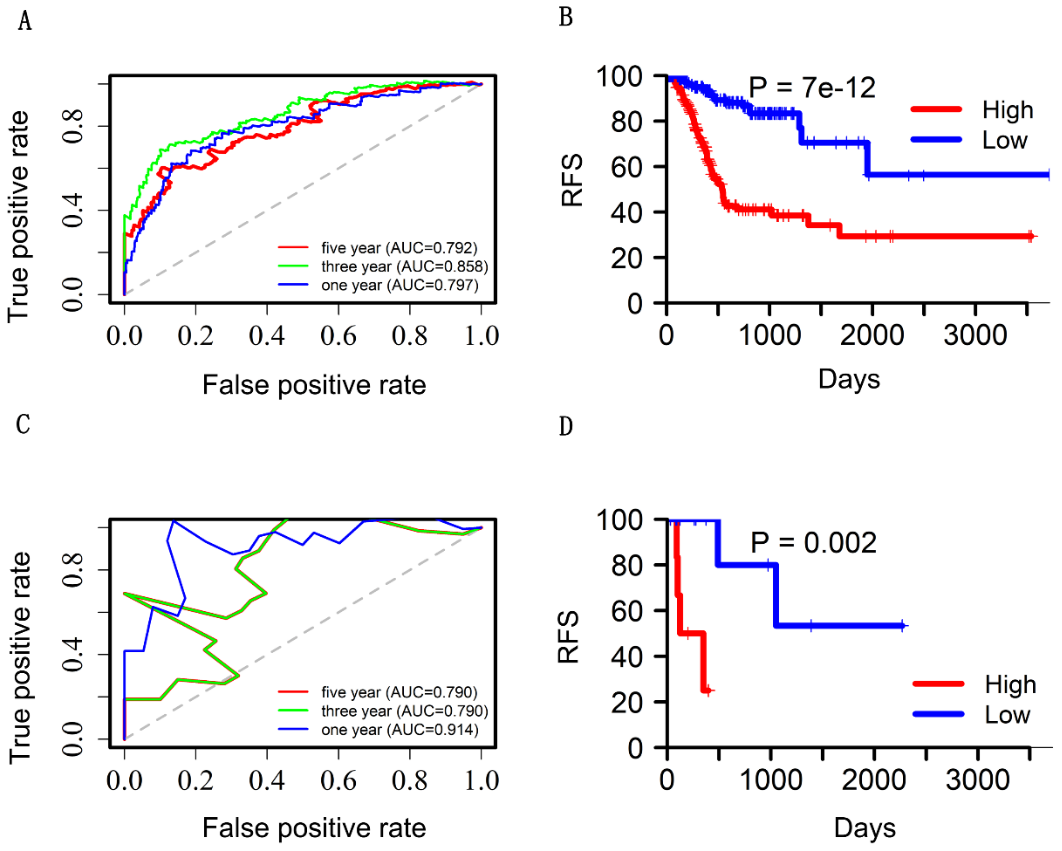

Table S1. The sequence of PCR primers and short hairpin RNA for MTA2.

Table S1. The sequence of PCR primers and short hairpin RNA for MTA2.	
Oligo Set	Sequences
MTA2	5'-CTCCTCGTCTCCCGGTTC-3' (Forward)
	5'-GTAAAACGACGGCCAGT-3' (Reverse)
MCM5	5′-AGCATTCGTAGCCTGAAGTCG-3′ (Forward)
	5′-CGGCACTGGATAGAGATGCG-3′ (Reverse)
shMTA2 #1	5-CCGGGCTCAACAAGACTGCAAATGGCTCGAGCCATTTGCAGTCTTGTTGAGCTTTTTG-3′ (F)
	5-AATTCAAAAAGCTCAACAAGACTGCAAATGGCTCGAGCCATTTGCAGTCTTGTTGAGC-3′ (R)
shMTA2 #1	5-CCGGGCAGATCGACCAGTTTCTTGTCTCGAGACAAGAAACTGGTCGATCTGCTTTTTG-3′ (F)
	5-AATTCAAAAAGCAGATCGACCAGTTTCTTGTCTCGAGACAAGAAACTGGTCGATCTGC -3′ (R)

Table S2. Clinical characteristics of included patients.

Characteristics	Total	Training dataset (n=269)	Testing dataset (n=115)	GSE26253 (n=432)
Age				
<65	155(40.36)	106(39.41)	49(42.61)	
>=65	229(59.64)	163(60.59)	66(57.39)	
Sex				
FEMALE	137(35.68)	99(36.8)	38(33.04)	
MALE	247(64.32)	170(63.2)	77(66.96)	
Histological_type				
Stomach Adenocarcinoma	213(55.49)	149(55.39)	64(55.65)	
Stomach- Intestinal Adenocarcinoma	170(44.27)	120(44.61)	50(43.48)	
Not Available	1(0.26)		1(0.87)	
Stage				
Stage I	56(14.58)	43(15.99)	13(11.3)	68(15.7)
Stage II	119(30.99)	80(29.74)	39(33.91)	167(38.7)
Stage III	144(37.5)	105(39.03)	39(33.91)	130(30.1)
Stage IV	42(10.94)	28(10.41)	14(12.17)	67(15.5)
Stage X	23(5.99)	13(4.83)	10(8.7)	
Tumor				
T1	21(5.47)	14(5.2)	7(6.09)	
T2	88(22.92)	65(24.16)	23(20)	
T3	167(43.49)	118(43.87)	49(42.61)	
T4	100(26.04)	68(25.28)	32(27.83)	
TX	8(2.08)	4(1.49)	4(3.48)	
Node				
N1	105(39.03)	69(37.1)	36(43.37)	
N2	79(29.37)	55(29.57)	24(28.92)	
N3	66(24.54)	49(26.34)	17(20.48)	
NX	19(7.06)	13(6.99)	6(7.23)	
Metastasis status				

M0	339(88.28)	239(88.85)	100(86.96)	
M1	26(6.77)	18(6.69)	8(6.96)	
MX	19(4.95)	12(4.46)	7(6.09)	
Cancer status				
TUMOR FREE	262(68.23)	184(68.4)	78(67.83)	
WITH TUMOR	69(17.97)	49(18.22)	20(17.39)	
Not Available	53(13.80)	36(13.38)	17(14.78)	
Ethnicity				
HISPANIC OR LATINO	5(1.3)	2(0.74)	3(2.61)	
NOT HISPANIC OR LATINO	274(71.35)	198(73.61)	76(66.09)	
Not Available	105(27.35)	69(25.65)	36(31.3)	
Residual tumor				
R0	311(80.99)	218(81.04)	93(80.87)	
R1	13(3.39)	10(3.72)	3(2.61)	
R2	18(4.69)	13(4.83)	5(4.35)	
RX	19(4.95)	12(4.46)	7(6.09)	
Not Available	23(5.99)	16(5.95)	7(6.09)	
Race				
ASIAN	77(20.05)	52(19.33)	25(21.74)	
BLACK OR AFRICAN AMERICAN	12(3.12)	9(3.35)	3(2.61)	
NATIVE HAWAIIAN OR OTHER PACIFIC ISLANDER	1(0.26)	1(0.37)		
WHITE	239(62.24)	171(63.57)	68(59.13)	
Not Available	55(14.33)	36(13.38)	19(16.52)	
Grade				
G1	10(2.6)	7(2.6)	3(2.61)	
G2	144(37.5)	102(37.92)	42(36.52)	
G3	222(57.81)	153(56.88)	69(60)	
GX	8(2.08)	7(2.6)	1(0.87)	
h_pylori_infection				
No	141(36.72)	96(35.69)	45(39.13)	
Yes	19(4.95)	12(4.46)	7(6.09)	
Not Available	224(58.33)	161(59.85)	63(54.78)	

Number node				
0	99(25.78)	72(26.77)	27(23.48)	
1_2	77(20.05)	53(19.7)	24(20.87)	
>2	165(42.97)	115(42.75)	50(43.48)	
Not Available	43(11.2)	29(10.78)	14(12.17)	
Barretts esophagus				
No	198(51.56)	129(47.96)	69(60)	
Yes	20(5.21)	18(6.69)	2(1.74)	
Not Available	166(43.23)	122(45.35)	44(38.26)	
Antireflux treatment				
NO	134(34.9)	93(34.57)	41(35.65)	
YES	42(10.94)	25(9.29)	17(14.78)	
Not Available	208(54.17)	151(56.14)	57(49.56)	

Table S3. Hazard ratios and 95% CIs as well as P.values of 721 TFs based on univariate Cox regression analysis.

Characteristics	Hazard.Ratio	CI95	P.value
NR1D1	1.00025	0.99991-1.00058	0.15547
HOXA11	0.99887	0.99723-1.00052	0.18087
HOXA10	0.99986	0.99923-1.0005	0.66665
DDIT3	1.00016	1.00001-1.00031	0.04032
BTG2	0.99999	0.99994-1.00003	0.55033
FOXN1	0.9995	0.9976-1.00141	0.61055
ZNF300	1.00185	0.99969-1.00402	0.09293
HOXB13	0.99957	0.99875-1.0004	0.31092
TIAL1	0.99989	0.9994-1.00038	0.66654
FUBP3	0.99933	0.99872-0.99993	0.02997
FUBP1	1.00005	0.99969-	0.80198

		1.0004	
BRIP1	0.99885	0.9974-1.00031	0.12148
ENO1	0.99999	0.99997-1.00002	0.54596
EOMES	1.00048	0.99885-1.00212	0.56118
ZBTB7A	0.99987	0.99972-1.00002	0.08646
ILF3	0.99997	0.99982-1.00011	0.63649
ILF2	0.99997	0.99983-1.0001	0.62947
LMX1B	0.99983	0.99814-1.00151	0.84058
GBX2	0.99482	0.98388-1.00588	0.35755
SETBP1	1.00013	0.99978-1.00047	0.46538
POU3F1	1.00041	0.99486-1.006	0.88426
DMAP1	1.00011	0.99877-1.00145	0.87289
XRCC5	0.99992	0.99979-1.00006	0.26154
XRCC6	0.99982	0.99967-0.99997	0.01651
MEIS2	1.00037	0.99991-1.00082	0.11584
MEIS1	1.00005	0.99973-1.00037	0.75909
HES1	1.00002	0.99988-1.00017	0.74829
BCL6	1.0002	0.99994-1.00046	0.12813
ASXL1	1.00019	1.00001-1.00036	0.03326
HIRA	0.99808	0.99663-0.99954	0.01007
CRABP2	1.00003	0.99992-1.00013	0.62258
ZNF410	1.00433	0.99478-1.01398	0.37516
TFAP4	0.99926	0.99824-	0.15748

		1.00029	
EAF1	0.99935	0.99849-1.0002	0.13279
FOXF1	1.00003	0.99985-1.00022	0.74673
FOXF2	1.00008	0.99978-1.00038	0.61463
PDX1	0.99986	0.99931-1.00042	0.6283
GABPB2	0.99987	0.99921-1.00053	0.69798
NOTCH3	1.00006	1.00001-1.00012	0.02022
ZNF383	1.00018	0.99839-1.00197	0.84571
ZNF382	1.00156	0.99846-1.00467	0.32515
EHMT2	0.99994	0.99971-1.00018	0.63533
HTATIP2	0.99993	0.9996-1.00025	0.6541
NFYC	0.99989	0.99925-1.00054	0.74221
NFYB	1.00028	0.99929-1.00127	0.58284
NFYA	0.99988	0.99945-1.00031	0.58329
SKIL	1.00023	1.00008-1.00038	0.00221
THRB	1.00026	0.99984-1.00069	0.22865
THRA	0.99998	0.99987-1.00009	0.66364
CREB5	1.00035	0.99941-1.00129	0.46346
CREB1	1.00037	0.99961-1.00113	0.33925
AES	0.99997	0.99989-1.00006	0.54069
LHX2	0.99898	0.98809-1.00998	0.85481
LHX4	1.00294	0.99619-1.00974	0.39425

YBX1	0.99993	0.99988-0.99999	0.02288
NCOA2	0.99987	0.99961-1.00013	0.3444
NCOA3	1.00013	0.99997-1.00029	0.11557
NCOA1	1.00014	0.99983-1.00045	0.37045
NCOA6	1.00003	0.99986-1.00021	0.70396
ERF	1.00008	0.99982-1.00035	0.52813
ERG	1.00041	0.99949-1.00132	0.38607
HAND2	0.99995	0.99975-1.00014	0.5863
HOXA7	1.00064	0.9967-1.00459	0.75163
HOXA5	1.00053	0.99947-1.00159	0.32395
HOXA4	1.00099	0.99941-1.00258	0.21949
HOXA1	1.00269	1.00061-1.00478	0.01142
WWP1	0.99977	0.99943-1.00011	0.19215
JARID2	1.00023	0.99981-1.00066	0.28573
NFKBIB	1.00023	0.9998-1.00066	0.29509
NFKBIA	1.00001	0.99986-1.00016	0.86283
NFKBIZ	1.00009	0.99989-1.00029	0.38508
NR5A1	1.00466	1.0001-1.00924	0.04528
DLX5	0.99984	0.99809-1.0016	0.86221
DLX3	0.99882	0.99718-1.00046	0.15786
TP63	0.99991	0.99956-1.00027	0.62796
WWTR1	1.00007	0.99998-1.00016	0.10785

CNBP	0.99999	0.99986-1.00012	0.88126
PIR	0.99905	0.99788-1.00022	0.11063
MLXIPL	0.9998	0.99953-1.00007	0.14937
HNRNPR	0.99977	0.99952-1.00003	0.08075
MAML1	1.0001	0.99966-1.00055	0.64435
TEAD1	1.00004	0.9999-1.00019	0.56026
TEAD4	0.99969	0.99896-1.00042	0.40773
KDM2A	1.00009	0.99991-1.00027	0.32435
FOXM1	1	0.99981-1.00019	0.99924
UBTF	1	0.99957-1.00043	0.99645
RELB	0.99997	0.99945-1.0005	0.91434
RELA	1.00014	0.99982-1.00046	0.40594
HDGF	0.99998	0.99993-1.00004	0.58959
COPS5	0.99981	0.99928-1.00034	0.47876
SSB	0.99995	0.99965-1.00025	0.74574
ZNF335	1.00037	0.99985-1.00088	0.16653
PRDM2	0.99987	0.99936-1.00037	0.60335
EGR2	1.00001	0.99971-1.00032	0.93016
EGR3	1.00009	0.99961-1.00057	0.7116
EGR1	1.00001	0.99998-1.00003	0.59844
ZEB1	1.00004	0.99992-1.00015	0.50717
AATF	0.99994	0.99965-1.00022	0.66829

NFKB1	0.9997	0.99927-1.00013	0.1719
NFKB2	0.99999	0.99978-1.00019	0.89309
HOPX	1	0.99988-1.00012	0.98877
NFATC1	1.00017	0.9998-1.00053	0.36513
NFATC2	1.00026	0.99998-1.00053	0.07329
NRIP1	0.99996	0.99953-1.0004	0.86406
FOS	1	0.99997-1.00003	0.9656
SEC14L2	1.0003	0.99939-1.00121	0.51773
UPF1	1.00003	0.99985-1.00021	0.74944
UPF2	0.99986	0.9995-1.00023	0.45854
ISL1	1.00083	0.99952-1.00214	0.21522
ZNF143	1.00058	0.99893-1.00223	0.4906
ZNF148	1.0002	0.99972-1.00067	0.40989
NANOG	1.00839	0.99957-1.0173	0.06242
LYL1	1.00029	0.99926-1.00131	0.58175
STAT5B	1.00009	0.99978-1.0004	0.5739
CREB3L1	1	0.99998-1.00002	0.78254
CTCF	1.0001	0.99956-1.00065	0.70708
ZNF423	1.00073	1.00024-1.00123	0.00361
NUPR1	0.99998	0.99977-1.00019	0.83857
SKI	1.00013	0.99998-1.00028	0.09623
PHF8	1.00032	0.99975-1.0009	0.27232

ATRX	1.00005	0.99991-1.00019	0.49133
PREB	0.99964	0.99917-1.00011	0.13473
VHL	0.99989	0.99941-1.00038	0.66041
CEBPZ	0.99957	0.99894-1.00019	0.17246
APBB1	1.00029	0.9998-1.00077	0.24599
WDR5	0.9991	0.99857-0.99964	0.00099
POU2AF1	0.99999	0.99986-1.00012	0.88648
ZFHX3	1.00012	0.99996-1.00028	0.15526
CBX8	1.00018	0.99824-1.00212	0.85903
CBX7	1	0.99971-1.0003	0.99362
ERCC2	1.00033	0.99938-1.00128	0.49361
CREG1	1.00002	0.99988-1.00016	0.79704
ABL1	1	0.99986-1.00015	0.98752
HNRNPD	0.99995	0.99982-1.00009	0.51723
CTBP1	0.99991	0.99969-1.00013	0.42549
EWSR1	0.99982	0.99963-1.00002	0.08372
DNMT1	1.00002	0.99982-1.00022	0.86237
HOXD9	1.00139	0.99864-1.00414	0.3212
HOXD3	1.02882	0.99326-1.06566	0.11338
KLF7	1.00018	0.99978-1.00057	0.3781
KLF6	1.00004	0.99996-1.00012	0.35782
KLF5	0.99998	0.99994-1.00003	0.40768

KLF4	0.99993	0.99982-1.00004	0.21396
KLF3	0.99978	0.99961-0.99995	0.01141
KLF2	0.99998	0.99988-1.00007	0.60479
KLF1	1.02317	1.00249-1.04429	0.02793
KLF9	1.00001	0.99989-1.00013	0.82379
KLF8	0.99994	0.99884-1.00104	0.91531
REST	1.00001	0.99945-1.00058	0.9589
POU1F1	1.09927	1.02023-1.18444	0.01292
RARA	1	0.99993-1.00007	0.95989
RARB	1.00295	1.00136-1.00455	0.00029
RARG	0.99996	0.99969-1.00022	0.75442
NRL	1.00283	0.98937-1.01648	0.68173
SALL4	1.00023	0.99965-1.00081	0.44047
HBP1	1.00003	0.99945-1.00062	0.91023
MXI1	0.99977	0.9993-1.00023	0.3202
MEN1	0.99986	0.99938-1.00034	0.5746
AHR	1.00003	0.99995-1.0001	0.45727
UHRF1	0.99966	0.99906-1.00027	0.27725
RB1	0.99996	0.99973-1.0002	0.75136
FHL2	0.99998	0.99987-1.0001	0.7695
PAWR	0.99999	0.9996-1.00038	0.95546
TP53BP1	1.00039	0.99977-1.00101	0.22026

DDB2	0.99917	0.99834-1	0.05034
EHF	0.99983	0.9997-0.99997	0.01534
ARNTL	0.99943	0.99772-1.00114	0.5159
MYBBP1A	1.00002	0.99973-1.0003	0.91515
HDAC1	0.99991	0.99974-1.00007	0.27319
HDAC3	1.00002	0.99911-1.00093	0.96315
HDAC2	0.99994	0.99971-1.00018	0.63987
HDAC5	1.00029	1.00001-1.00057	0.04447
HDAC4	0.99974	0.99938-1.00009	0.14808
HDAC7	1.00049	1.00016-1.00082	0.00365
HDAC9	1.00003	0.99965-1.00041	0.8884
SRCAP	1.00015	1.00005-1.00026	0.00331
CEBPB	1.00008	0.99997-1.00018	0.16023
CEBPA	1.00004	0.99992-1.00016	0.49649
CEBPG	0.9998	0.99944-1.00016	0.28071
CEBPE	1.00443	0.9772-1.03242	0.75249
CEBPD	1.00002	0.99993-1.0001	0.69132
HDAC11	0.99948	0.99856-1.00041	0.27235
HIF3A	1.0001	0.99993-1.00028	0.2405
XPC	0.9998	0.99881-1.00079	0.68719
DENND4A	1.00036	0.99973-1.001	0.26426
TRERF1	1.00007	0.99956-1.00058	0.77893
SMAD4	1.00002	0.99956-	0.94578

		1.00047	
SMAD7	1.00061	1.00007-1.00116	0.02583
SMAD1	0.99884	0.99726-1.00042	0.14965
SMAD2	0.99974	0.99936-1.00013	0.19459
SMAD3	1.00029	1.00013-1.00045	0.00033
RFX5	0.99989	0.9995-1.00027	0.56961
RFX1	1.00045	0.99956-1.00134	0.32244
RFX2	1.00033	0.99971-1.00095	0.30255
RFX3	1.0002	0.99913-1.00127	0.71504
APC	0.99986	0.99936-1.00036	0.58129
CDC5L	0.9997	0.9993-1.0001	0.14608
BRD7	0.99996	0.99958-1.00035	0.85769
SLA2	0.99995	0.9978-1.0021	0.96118
NEUROG3	0.99911	0.99696-1.00127	0.4181
GTF2F1	0.9999	0.99943-1.00036	0.66234
CUX1	1.00002	0.99991-1.00013	0.75127
TOB1	0.99997	0.99981-1.00013	0.70621
EVX1	0.99903	0.99709-1.00098	0.32904
CTNNBIP1	0.99938	0.99874-1.00003	0.06068
CITED2	0.99991	0.9997-1.00013	0.43033
EBF3	1.00134	0.99837-1.00431	0.37716
EBF1	1.00009	0.99972-1.00046	0.63439
FOXH1	0.99962	0.99654-1.00272	0.81177

HLTF	1.0002	0.99985-1.00056	0.26158
RUNX1T1	1.00029	0.99977-1.00082	0.27342
TFAP2A	0.99996	0.99962-1.0003	0.81353
TFAP2C	0.99965	0.99868-1.00062	0.48137
RREB1	0.99998	0.99974-1.00021	0.84645
CDCA7L	1.00002	0.99955-1.00048	0.94753
ZHX2	1.00049	0.99992-1.00107	0.09331
DEK	0.99993	0.99979-1.00006	0.26068
TBPL1	0.99899	0.9975-1.00048	0.18215
FUS	1.00012	0.99997-1.00027	0.11263
ONECUT2	1.00009	1.00002-1.00016	0.01481
ONECUT1	1.00318	1.00119-1.00518	0.00173
HSF1	0.99994	0.99975-1.00014	0.58759
HSF2	0.99933	0.99732-1.00134	0.51302
HSF4	0.99986	0.99782-1.0019	0.89098
RORC	0.99973	0.99916-1.00031	0.35927
RORA	1.00014	0.99955-1.00073	0.64572
TRRAP	1.00002	0.99997-1.00007	0.39995
ZMYND11	0.99992	0.99962-1.00022	0.61658
TAF5	0.99816	0.99495-1.00137	0.26101
TAF1	0.99971	0.99917-1.00025	0.28887
FOSB	1	0.99996-1.00004	0.91782

PDCD11	0.99993	0.99955-1.00031	0.7306
LIN28A	1.00606	1.0012-1.01093	0.01441
CREM	1.00049	0.99949-1.0015	0.3357
TRIM28	1.00005	1-1.0001	0.07447
TRIM22	1.00007	0.99989-1.00026	0.43034
GTF3A	0.99993	0.99978-1.00009	0.38464
SHOX	1.00736	0.99945-1.01534	0.06835
IFI16	1.00014	0.99999-1.0003	0.0723
LRRFIP1	1.0001	0.99996-1.00025	0.15447
POU3F2	1.00178	1.00024-1.00332	0.02332
SP100	1.00025	0.99998-1.00051	0.06668
ZNF217	1.00019	1.00006-1.00031	0.003
NR2E3	1.0241	1.00534-1.04322	0.0116
SMG6	0.99995	0.99893-1.00097	0.91787
SF1	0.99995	0.99974-1.00016	0.61368
SCD5	1	0.99941-1.0006	0.9879
ETS2	0.9999	0.99983-0.99998	0.00997
ETS1	1.00011	0.99999-1.00023	0.0717
CBFA2T3	1.0002	0.99953-1.00086	0.55838
JUN	0.99999	0.99994-1.00004	0.7594
NPAS3	1.00252	1.00006-1.00498	0.04452
NPAS2	1.00001	0.99981-1.00021	0.90265

PURA	1.00004	0.99961-1.00048	0.84848
NFAT5	0.99993	0.99971-1.00014	0.51696
GCM1	1.00986	0.99641-1.02348	0.15138
MXD1	0.99995	0.99984-1.00005	0.32015
MED23	0.99989	0.99916-1.00061	0.75964
NONO	0.99995	0.99985-1.00005	0.30255
PARP1	0.99997	0.99983-1.00011	0.70098
TFDP1	0.9999	0.99971-1.00009	0.31111
ANKRD1	1.00044	0.99971-1.00118	0.23953
BIN1	1.00027	1.00001-1.00053	0.03971
FOXP2	1.00005	0.99974-1.00037	0.74972
FOXP3	1.00032	0.99888-1.00175	0.66478
FOXP1	1.00007	0.9999-1.00024	0.39149
MYCN	1.00014	0.99998-1.00031	0.08895
ECD	1.00054	1.00004-1.00105	0.03531
ARID3A	1.00009	0.99998-1.0002	0.09705
BTF3	0.99996	0.99989-1.00003	0.29326
FOXO3	1.00005	0.99988-1.00022	0.57181
FOXO1	1.00006	0.9997-1.00042	0.74996
FOXO4	0.99919	0.99812-1.00027	0.14165
PIAS2	0.99974	0.99876-1.00072	0.60572
PIAS3	1.00056	1.00016-1.00095	0.0054

PIAS1	1.00004	0.99962-1.00046	0.83731
PIAS4	0.99958	0.99853-1.00063	0.42913
HMGB2	0.99991	0.99973-1.0001	0.3547
BARD1	0.99876	0.99735-1.00018	0.08674
TTF2	0.99923	0.99848-0.99997	0.04262
POU5F1	1.00148	1.00009-1.00287	0.03657
ZNF160	1.00021	0.99943-1.00098	0.60388
GTF2A1	0.99986	0.99914-1.00057	0.69097
BPTF	1.00011	0.99988-1.00034	0.34328
TCF12	0.99982	0.99964-1	0.04778
TCF19	0.99978	0.99918-1.00037	0.46207
CREB3	0.99979	0.99934-1.00025	0.37377
ASCL1	1.00024	1.00008-1.00039	0.00352
ZFP36	1.00002	1-1.00003	0.10538
NEUROD1	1.00026	0.99994-1.00059	0.11152
TFCP2L1	0.99992	0.99967-1.00017	0.544
TFCP2	0.99978	0.99897-1.00058	0.58725
OTX1	0.99978	0.99832-1.00124	0.76726
YEATS4	0.99994	0.99944-1.00044	0.81641
HMGA2	1.00017	1.00004-1.00031	0.01359
HMGA1	1.00001	0.99998-1.00004	0.61528
HAX1	1.00008	0.99982-1.00034	0.55743
NELFB	0.9995	0.9991-0.9999	0.01347

NFRKB	0.99971	0.99905-1.00038	0.3981
DBP	1.00018	0.99945-1.0009	0.63615
MED1	0.99986	0.99959-1.00012	0.2869
TCF4	1.00012	0.99992-1.00033	0.22325
TCF7	0.99994	0.99971-1.00017	0.61466
TCF3	0.99996	0.99978-1.00014	0.6825
PCBD1	0.99989	0.99972-1.00006	0.21399
PITX2	1.00015	0.99781-1.0025	0.89926
PITX3	1.07771	1.03337-1.12394	0.00048
PITX1	1	0.99994-1.00005	0.94476
KAT2B	0.99994	0.99945-1.00043	0.81642
MAFK	1.00029	0.99992-1.00066	0.12723
MAFA	1.00394	0.99882-1.00908	0.13146
MAFB	1.00016	0.99987-1.00045	0.27362
MAFF	1.00003	0.99968-1.00037	0.88499
MAFG	0.99987	0.99949-1.00026	0.52209
RBPJ	0.99986	0.99954-1.00017	0.37511
TRAF6	0.99764	0.99585-0.99944	0.01006
POLR1A	0.99968	0.99935-1.00001	0.05446
NR1H4	1.00073	0.99942-1.00205	0.27516
NR1H2	1.00022	0.99989-1.00056	0.19231
HOXB7	0.9999	0.99929-1.00052	0.76049

HOXB4	1.00117	1.00005-1.00229	0.04042
SPDEF	0.99997	0.99971-1.00024	0.84517
NR2F6	1.00003	0.99986-1.00021	0.70965
NR2F1	1.00018	0.9999-1.00047	0.20251
SNAI2	1.00017	0.99938-1.00096	0.67895
SNAI1	1.00017	0.99865-1.00169	0.82878
HDAC10	1.00061	0.99893-1.00229	0.47739
SUGP1	0.99791	0.99583-1	0.0497
TP53	0.99981	0.99961-1.00002	0.07113
TRIB3	0.9999	0.99964-1.00016	0.46819
EZH2	0.99929	0.99856-1.00002	0.05741
NR4A2	0.99983	0.9996-1.00006	0.1512
NR4A3	0.99998	0.99974-1.00022	0.87435
NR4A1	0.99998	0.99992-1.00004	0.59032
NCOA4	0.99994	0.99983-1.00004	0.23745
HIC1	1.00032	0.99995-1.00069	0.08828
RXRA	0.99977	0.9995-1.00003	0.08748
ZNF267	1.00079	0.99966-1.00192	0.16993
RAD51	0.99922	0.99762-1.00083	0.34305
ZNRD1	0.99965	0.99856-1.00074	0.52555
ETV1	1.00054	1.00022-1.00086	0.00099
ETV3	0.99955	0.99872-1.00037	0.28045

ETV5	0.99999	0.9997-1.00027	0.92731
ETV4	0.99995	0.99975-1.00014	0.57834
ETV7	0.99982	0.9992-1.00044	0.57549
ETV6	0.99987	0.99954-1.00019	0.42256
IKZF1	1.00018	0.99994-1.00041	0.13419
BRPF1	1.00001	0.99881-1.00121	0.99277
SP1	0.99984	0.99966-1.00003	0.09778
SP2	1.00008	0.99922-1.00093	0.86015
SP3	1	0.99962-1.00038	0.99951
SP4	1.00091	0.99979-1.00203	0.11019
HMG20B	0.99962	0.99914-1.00011	0.1254
PBX2	1.00016	0.99992-1.0004	0.18587
PBX1	1	0.99985-1.00015	0.97563
AIRE	1.00192	0.99744-1.00641	0.40138
DR1	0.99974	0.99922-1.00027	0.33949
CHD4	1.00009	0.99997-1.00022	0.15325
CHD8	1.00016	0.99984-1.00049	0.33187
ZFP36L1	1.00004	0.99999-1.00008	0.11922
ATOH1	0.99968	0.99887-1.0005	0.44544
LIMD1	0.9998	0.99923-1.00037	0.48681
SPEN	0.99999	0.99978-1.0002	0.94205
DAXX	0.99991	0.99934-1.00047	0.73992

SHOX2	1.00454	1.00197-1.00712	0.00053
HIPK2	1.00002	0.99993-1.00012	0.63232
FOXJ1	1.00034	1.00002-1.00065	0.03476
TCFL5	0.99922	0.99815-1.00029	0.15143
RUVBL1	1.00018	0.99967-1.0007	0.48106
TBC1D22A	0.99984	0.99911-1.00058	0.67255
FOXL2	1.0004	0.9947-1.00614	0.88987
FOXL1	1.00086	1.0002-1.00152	0.01036
BRCA1	0.99974	0.99912-1.00035	0.40334
BRCA2	0.99992	0.99922-1.00063	0.83117
SMURF2	1.00041	0.99972-1.0011	0.2444
CTCFL	0.9996	0.99868-1.00051	0.38597
PCGF2	1.00023	0.99993-1.00053	0.12567
NR5A2	0.99982	0.99923-1.00042	0.56041
DLX4	0.99885	0.99684-1.00087	0.26604
HMGN1	0.99994	0.99977-1.00011	0.48841
MDM2	1.00001	0.99993-1.00009	0.777
MDM4	1.00024	0.99986-1.00061	0.21871
CRX	1.00343	0.99974-1.00713	0.06856
ARNTL2	1.00023	1.00002-1.00044	0.0324
GABPA	0.99944	0.99848-1.00041	0.25657
GLIS3	1.00037	1-1.00075	0.05214

SREBF2	0.99991	0.99981-1.00001	0.06644
SREBF1	0.99999	0.99992-1.00006	0.80619
MTA1	1.00017	0.99983-1.00051	0.32333
MTA2	0.99945	0.99914-0.99977	0.00062
MTA3	1.00036	0.99965-1.00107	0.31973
BACH2	1.0008	1.00019-1.0014	0.00981
KCNIP3	1.00058	0.99918-1.00199	0.41715
ELK3	1.00017	0.99991-1.00043	0.20716
ELK1	1.00005	0.99949-1.0006	0.87198
ELK4	1.00015	0.99977-1.00053	0.445
PER2	1.00011	0.99967-1.00056	0.61365
KAT5	0.99942	0.9983-1.00054	0.30945
PLAG1	1.0026	1.00128-1.00391	1.00E-04
JDP2	0.99913	0.99826-0.99999	0.04852
ATF4	0.99984	0.99971-0.99997	0.01281
ATF7	1.00018	0.99965-1.00072	0.50086
ATF6	1.00012	0.99973-1.00051	0.55579
ATF1	0.99894	0.9977-1.00018	0.09348
ATF3	1.00002	0.99986-1.00017	0.82894
ATF2	1.00041	0.99979-1.00103	0.19171
LCOR	1.00003	0.99972-1.00034	0.83893
TSC22D1	0.99997	0.99989-1.00006	0.53402

TSC22D3	1.00006	0.99997-1.00014	0.19075
TNFAIP3	1.00001	0.99986-1.00016	0.8817
GFI1	0.99992	0.99743-1.0024	0.94675
HINFP	0.99957	0.99773-1.00142	0.6496
REL	1.00021	0.99925-1.00116	0.67204
ZNF239	0.99944	0.99735-1.00154	0.60164
TAL1	1.00198	0.99955-1.00442	0.10986
FLI1	1.00025	0.99962-1.00089	0.43028
SIRT3	0.99921	0.99724-1.00118	0.42999
SIRT2	1.00011	0.99967-1.00055	0.6201
SIRT1	1.00048	0.99972-1.00123	0.21604
CTNNB1	0.99995	0.99989-1.00001	0.11268
GFI1B	1.0008	0.99941-1.00219	0.26125
NR2C2	1.00008	0.99963-1.00054	0.71789
MED15	0.99999	0.99989-1.0001	0.9122
TLX1	0.99983	0.99882-1.00084	0.73754
APEX1	0.99993	0.99976-1.00009	0.39859
ESRRA	0.99949	0.9991-0.99989	0.01134
ESRRB	1.00053	0.99449-1.00661	0.86341
BATF	0.9994	0.99821-1.0006	0.32886
HEXIM1	1.00017	0.99951-1.00083	0.60617
PML	1.0001	0.99998-1.00021	0.1075

DRAP1	1.00006	0.99977-1.00034	0.69994
TBR1	1.01068	1.00082-1.02064	0.0337
VEZF1	1.00007	0.99969-1.00045	0.71314
MEF2C	1.00011	0.99985-1.00037	0.40657
MEF2B	0.99937	0.99592-1.00284	0.72323
MEF2A	1.00012	0.99994-1.0003	0.1788
MEF2D	1.00011	0.99988-1.00034	0.35672
HIF1A	1	0.99993-1.00007	0.98205
PAX5	1.00016	1.00001-1.00032	0.04296
PAX6	0.99967	0.99811-1.00124	0.68198
PAX2	1.00096	0.99811-1.00381	0.51101
PAX8	1.00104	1.00032-1.00177	0.00454
RFXANK	0.99945	0.9985-1.0004	0.25265
NCOR1	0.99996	0.99987-1.00005	0.37914
TEF	0.99992	0.99929-1.00054	0.79242
LMO2	0.99986	0.9991-1.00061	0.71382
LMO3	1.00034	0.99966-1.00101	0.32471
LMO4	1.00007	1-1.00014	0.05039
UBP1	1	0.99963-1.00037	0.99722
PHB2	0.99989	0.99979-1	0.04099
PKNOX1	0.9986	0.99708-1.00012	0.07043
MCM5	0.99999	0.99975-1.00023	0.93569
MCM2	0.99996	0.99972-1.00021	0.7695

VDR	0.99979	0.99956-1.00001	0.06644
NF1	1.00011	0.9999-1.00032	0.29981
KLF10	1	0.99986-1.00014	0.95915
USF2	1.00005	0.99978-1.00032	0.70172
USF1	0.99975	0.999-1.00051	0.52083
RBMX	0.99988	0.99968-1.00009	0.26629
TCF7L2	1.00012	0.9998-1.00043	0.4656
CIITA	0.99999	0.99985-1.00014	0.94085
SOX11	1.00004	0.99716-1.00292	0.98086
SOX17	1.00038	0.99843-1.00234	0.70247
HES6	0.9994	0.99875-1.00006	0.07406
MITF	1.00027	0.99961-1.00094	0.4223
SUZ12	0.99987	0.99952-1.00023	0.4842
NFE2	0.99933	0.99642-1.00224	0.65067
FOXA1	1.00007	0.99991-1.00023	0.40995
FOXA3	0.99985	0.99955-1.00015	0.33536
FOXA2	1.00009	0.99985-1.00032	0.4709
NKX3_1	0.99982	0.99836-1.0013	0.81521
DACH1	0.99969	0.99912-1.00026	0.28719
TSG101	0.99997	0.99947-1.00047	0.90185
ZEB2	1.00011	0.99984-1.00038	0.41764
SNW1	0.9996	0.99879-1.0004	0.32463

TRIM16	1.00013	0.9995-1.00076	0.68579
URI1	1.00001	0.99997-1.00005	0.63641
SMARCB1	0.99972	0.99933-1.00012	0.16767
PPARGC1A	0.99991	0.99953-1.00029	0.62592
JUNB	1.00002	0.99998-1.00006	0.408
JUND	1	0.99995-1.00006	0.97999
PPARG	1.00003	0.99971-1.00034	0.85942
PPARD	1.00005	0.99982-1.00027	0.69581
PPARA	0.9996	0.99923-0.99998	0.03751
DDB1	0.99997	0.99985-1.00009	0.64396
DEDD	1.0004	0.99936-1.00144	0.45454
IKBKB	0.99966	0.99892-1.00041	0.37494
PLAGL2	1.00003	0.99991-1.00015	0.65499
PLAGL1	1.0001	1.00002-1.00018	0.01181
EPAS1	1.00007	0.99998-1.00015	0.12526
E2F7	0.99918	0.9978-1.00056	0.24359
ZNF202	0.99906	0.99696-1.00115	0.37746
ELL	1.00031	0.99919-1.00144	0.5845
NPM1	0.99997	0.99991-1.00002	0.23881
NAB1	0.99998	0.99974-1.00021	0.85153
NAB2	1.00089	1.00017-1.00162	0.01603
TP73	0.99984	0.99835-1.00133	0.83653

E4F1	1.0002	0.99896-1.00145	0.74967
ESRRG	1.00004	0.99958-1.0005	0.86938
ZBTB2	0.99962	0.99809-1.00116	0.63095
ZBTB5	0.99979	0.99902-1.00056	0.59301
TRPS1	1.00013	0.99978-1.00049	0.46515
GRHL1	0.99988	0.99951-1.00025	0.52027
NR3C2	0.99952	0.99883-1.00022	0.18046
NR3C1	1.00027	0.99997-1.00057	0.07367
NFE2L2	0.99985	0.99968-1.00002	0.07653
NFE2L1	1	0.99987-1.00013	0.96231
SCAND1	1.0001	0.9998-1.00039	0.52616
MLLT10	1.00005	0.99931-1.0008	0.88946
SOX9	1	0.99995-1.00005	0.95053
SOX2	1.00011	0.99998-1.00024	0.09689
SOX4	1.00004	0.99998-1.00009	0.1815
SOX5	1.00218	1.00033-1.00403	0.02099
CRTC1	1.00053	0.99967-1.00139	0.22606
BTAF1	1.00004	0.9996-1.00048	0.8512
MSC	1.00103	1.00021-1.00186	0.01368
NR1H3	0.99983	0.99917-1.00049	0.61252
SLC2A4RG	1.00004	0.99991-1.00018	0.52292
RFXAP	0.99856	0.99537-1.00175	0.37513

ELF1	1.00005	0.99982-1.00027	0.67454
ELF3	0.99999	0.99994-1.00004	0.76761
ELF2	1.00048	0.99956-1.00141	0.30637
ELF4	1.00002	0.99978-1.00027	0.86583
AIP	1.00031	0.99987-1.00075	0.16877
RNF14	1.00074	0.99999-1.00149	0.05378
FOXQ1	1	0.99989-1.0001	0.93789
HLX	1.00157	0.99948-1.00366	0.14191
HLF	0.99991	0.99951-1.00032	0.6682
SIX1	1	0.99864-1.00136	0.99766
KHDRBS1	0.99978	0.99948-1.00008	0.15789
MYBL1	1.00009	0.99894-1.00123	0.88192
MYBL2	1.00007	0.99997-1.00017	0.17979
SRSF1	0.99982	0.99958-1.00006	0.13939
SRF	0.99995	0.99978-1.00011	0.50995
EIF2AK2	1.00003	0.99985-1.0002	0.77637
HNF4G	0.99985	0.99964-1.00007	0.1823
HNF4A	0.99997	0.99988-1.00006	0.49472
MLX	0.99966	0.99923-1.00009	0.12272
DEAF1	0.99985	0.999-1.0007	0.72297
MECP2	0.99997	0.99964-1.00029	0.84111
GZF1	0.99972	0.99858-1.00086	0.63007

ATM	1.00018	0.9999-1.00047	0.21157
TBX21	0.99857	0.99186-1.00533	0.67818
KLF13	0.99981	0.99963-0.99999	0.04022
KLF12	1.00035	1.00006-1.00064	0.01885
KLF11	1.00007	0.99928-1.00087	0.85296
KLF16	0.99986	0.99946-1.00025	0.47102
KLF15	1.00032	0.99929-1.00136	0.53995
HHEX	1.00046	1.00006-1.00085	0.02273
DNMT3A	1.00035	0.99988-1.00082	0.14182
ZNF175	1.0018	0.99953-1.00408	0.12001
NFIL3	1.00006	0.99941-1.0007	0.86318
PTTG1	0.9998	0.99939-1.00022	0.35516
E2F6	0.99902	0.99619-1.00187	0.5007
E2F5	0.99971	0.99858-1.00085	0.61903
E2F4	1.00013	0.99978-1.00048	0.47074
E2F3	1.00016	0.99932-1.00101	0.70618
E2F2	0.99881	0.99776-0.99986	0.02584
E2F1	0.99989	0.99935-1.00044	0.70064
E2F8	0.99898	0.99815-0.99981	0.01645
TBP	0.99744	0.995-0.99989	0.0403
RBBP7	0.99983	0.99962-1.00005	0.13
FOXD3	1.00176	0.99409-1.00948	0.65386

MLLT3	0.99964	0.99878-1.0005	0.41269
CDX1	0.99981	0.99965-0.99998	0.02629
CDX2	0.99978	0.99956-1.00001	0.05552
NKX2_3	1.00056	0.99709-1.00404	0.7536
NKX2_5	0.9999	0.99855-1.00124	0.87912
TLE3	1.00005	0.99979-1.00031	0.69122
TFPT	1.00129	1.00006-1.00252	0.03911
MBD1	0.99985	0.99925-1.00046	0.63622
MBD2	0.99974	0.99938-1.0001	0.16376
IRF3	1.00022	0.9998-1.00064	0.31038
IRF1	0.99991	0.99979-1.00003	0.13953
IRF7	1.00022	0.99994-1.0005	0.1212
IRF6	0.99993	0.99971-1.00015	0.53352
IRF4	0.99989	0.99939-1.00038	0.64878
IRF9	0.99988	0.99791-1.00186	0.90628
IRF8	1.00007	0.99984-1.00031	0.55196
ZBTB17	0.99989	0.99864-1.00114	0.85835
ZBTB16	0.99998	0.99933-1.00063	0.9445
ZBTB14	1.0001	0.99784-1.00236	0.934
NRF1	0.99898	0.9957-1.00227	0.54183
SERTAD1	1.00024	0.99941-1.00107	0.57777
FOSL1	1.0002	1.0001-1.00031	0.00014

FOSL2	0.99996	0.99988-1.00004	0.30138
MAX	0.99917	0.99853-0.99981	0.01079
MAZ	1.0001	0.9999-1.00031	0.33392
MAF	0.99998	0.99978-1.00019	0.8751
MAL	0.99999	0.99995-1.00002	0.55498
IRF2	0.99958	0.999-1.00016	0.15367
IRF5	1.00111	1.00014-1.00209	0.02444
TBL1X	1.00002	0.99968-1.00037	0.90492
SMARCA1	1.00038	1.00004-1.00073	0.02912
SMARCA4	1.00001	0.99986-1.00016	0.8821
PA2G4	0.99991	0.99977-1.00005	0.22649
NR1I3	1.00927	0.99875-1.0199	0.0845
NR1I2	0.99975	0.99922-1.00028	0.35364
HOXC9	1.001	0.99937-1.00264	0.22906
HOXC8	1.00035	0.99828-1.00242	0.7416
HOXC6	1.00049	0.999-1.00199	0.51848
MSX1	0.99916	0.99781-1.00051	0.21994
MSX2	0.99984	0.99946-1.00022	0.4143
SIN3A	1.00025	0.99991-1.00059	0.14812
ZIC2	0.99968	0.99893-1.00044	0.40702
ING4	1.00034	0.99909-1.00159	0.59832
ING1	1.00024	0.99907-1.00142	0.6832
ING2	0.9988	0.99604-	0.3947

		1.00156	
SNIP1	0.99888	0.99628-1.00148	0.39711
NCOR2	1.00004	0.99998-1.00011	0.18581
SUPT3H	1.00145	0.99874-1.00417	0.29498
RB1CC1	1.00006	0.99972-1.0004	0.73614
TFEB	0.99988	0.99929-1.00046	0.67777
LEF1	1.00026	0.99987-1.00064	0.19181
PGR	1.00013	0.99909-1.00116	0.81014
BMI1	1.00021	0.99983-1.00058	0.2845
CBFB	1.00028	0.99987-1.0007	0.18249
ZNF24	0.99988	0.99952-1.00024	0.51033
ARNT	1.00009	0.99956-1.00062	0.75196
RUNX2	1.00039	0.99987-1.00092	0.1441
RUNX3	1.00003	0.99968-1.00037	0.88118
ESR2	1.00344	0.9989-1.00801	0.13764
ESR1	0.99964	0.99804-1.00125	0.66141
TBX2	1.00029	0.9999-1.00068	0.15148
TBX3	0.99969	0.99932-1.00006	0.10357
TBX5	1.00021	0.99772-1.00271	0.86835
BDP1	1.00018	0.9997-1.00066	0.45976
RFWD2	1.00037	0.99981-1.00094	0.19834
AR	1.00025	0.99916-1.00135	0.64847

CREB3L4	1.00032	0.99946-1.00119	0.46125
CNOT8	0.99975	0.99899-1.0005	0.51226
CNOT7	1.00003	0.99984-1.00022	0.75282
EAPP	0.99988	0.99925-1.00052	0.71558
EGF	1.00177	0.99851-1.00504	0.28877
NELFCD	0.99995	0.99979-1.00012	0.57422
KDM4B	1.00001	0.99941-1.00061	0.97402
WT1	1.00088	0.99842-1.00334	0.48527
GSC	1.00215	0.99632-1.00801	0.47076
NFATC3	1.00038	0.99968-1.00109	0.28681
ID4	1.00008	0.99956-1.00059	0.77362
ID2	0.99992	0.9997-1.00014	0.4691
ID3	0.99999	0.99978-1.00021	0.95475
ID1	1.00001	0.99993-1.0001	0.74539
GLI2	1.00057	0.99999-1.00116	0.05583
GLI3	1.00013	0.99958-1.00067	0.652
GLI1	1.00222	1.001-1.00344	0.00035
NR0B2	1.00002	0.99962-1.00042	0.92012
BARX2	0.99993	0.99952-1.00035	0.74532
HIVEP2	1.00041	1.00011-1.00071	0.00729
MYOCD	0.99998	0.99981-1.00015	0.83013
SFPQ	0.99988	0.9997-1.00005	0.17132

FOXK2	1.00013	0.99976-1.0005	0.49178
HCFC1	1.00002	0.9999-1.00014	0.79393
ZNF350	0.9993	0.99757-1.00104	0.43073
TWIST1	1.00021	0.99841-1.00201	0.81919
TWIST2	1.00034	0.99892-1.00176	0.63698
HNF1B	1.00009	0.99989-1.0003	0.35823
HNF1A	0.99994	0.99926-1.00062	0.86902
POU2F2	1.00001	0.99991-1.00011	0.79266
POU2F1	1.00022	0.99989-1.00055	0.18802
PRDM1	0.99997	0.99975-1.00018	0.76501
MYC	0.99991	0.99979-1.00002	0.09475
MYB	0.99937	0.99894-0.99979	0.00329
GATA6	0.99994	0.99982-1.00006	0.34862
GATA4	1.00002	0.99997-1.00007	0.45343
GATA5	1.00027	0.99998-1.00056	0.06898
GATA2	0.99985	0.99823-1.00147	0.85301
GATA3	1.00026	0.9992-1.00132	0.63445
GATA1	1.02245	0.972-1.07551	0.38982
CLOCK	0.99981	0.99937-1.00024	0.38361
KAT6A	0.99999	0.9997-1.00029	0.95661
BHLHE41	1.00016	1.00006-1.00026	0.0011
KDM4C	0.99902	0.99755-1.0005	0.1939

STAT5A	1.00005	0.99958-1.00051	0.84902
ZNF444	1.00043	1.00009-1.00077	0.01299
L3MBTL1	1.0008	0.99961-1.00199	0.18731
FOXC2	1.00376	1.00159-1.00595	7.00E-04
FOXC1	0.99998	0.99976-1.0002	0.87455
MZF1	1.00086	1.0001-1.00162	0.02673
HR	0.99989	0.99973-1.00005	0.17916
PROX1	0.99991	0.99971-1.0001	0.34861
GTF2B	0.99916	0.99812-1.00021	0.11727
GTF2I	0.99998	0.99969-1.00027	0.88419
CREBBP	1.00014	0.99998-1.0003	0.07712
HEYL	1.00031	1.0001-1.00052	0.00336
RBL1	0.99963	0.99878-1.00049	0.39835
RBL2	0.99999	0.99964-1.00035	0.97453
HEY2	0.99924	0.99619-1.00231	0.6279
HEY1	0.99963	0.99775-1.00151	0.69866
MTF1	0.99846	0.99722-0.9997	0.01463
ZNF224	0.99965	0.99795-1.00136	0.68786
HOXC11	0.99952	0.99827-1.00076	0.44553
HOXC10	1.00009	0.99966-1.00053	0.67553
HOXC13	0.9999	0.99842-1.00137	0.89027
SPI1	1.00005	0.99974-1.00035	0.76439

SPIB	1.00017	0.99997-1.00037	0.1033
SPIC	1.02418	1.01026-1.03829	0.00062
SIM2	0.99991	0.99957-1.00025	0.60395
LDB1	1.00014	0.99971-1.00057	0.51608
SATB2	0.99951	0.99872-1.0003	0.22368
SATB1	1.00023	0.99988-1.00057	0.19725
PHF10	0.99987	0.99946-1.00029	0.55524
BCL11A	1.00006	0.99958-1.00054	0.79963
POU2F3	1.00019	0.99923-1.00114	0.70375
ARID1B	1.00017	0.99994-1.00041	0.14918
ARID1A	1.0001	0.99994-1.00026	0.20847
MKL1	1.00067	1.0002-1.00113	0.00489
NHLH2	1.00246	0.99873-1.00621	0.19581
SND1	0.99998	0.99984-1.00011	0.72745
TGIF1	0.99993	0.99967-1.0002	0.62126
STAT6	0.99997	0.99985-1.00009	0.64767
STAT4	1.00051	0.998-1.00302	0.69207
STAT3	1.00008	0.99999-1.00016	0.06711
STAT2	1.00014	0.99989-1.0004	0.27866
STAT1	1	0.99996-1.00004	0.99538
ARX	1.00105	1.00011-1.00199	0.02858
ZNF76	1.00067	0.99969-1.00166	0.18086

NFIC	1.00001	0.99995-1.00006	0.80889
NFIB	1	0.9999-1.0001	0.99057
NFIA	1.00001	0.99988-1.00014	0.89744
NFIX	0.99994	0.99985-1.00003	0.1764

Table S4. Univariate Cox regression analysis and multivariate Cox regression analysis outcome based on TFs risk score and other clinical factors.

	Univariate Cox analysis				Multivariate Cox analysis			
Characteristics	HR	HR.95L	HR.95H	pvalue	HR	HR.95L	HR.95H	pvalue
Score	2.718282	2.25954	3.270159	2.87E-26	2.105799	1.724836	2.570906	2.60E-13
Sex	1.874275	1.183928	2.967164	0.007355	1.416686	0.883074	2.272744	0.148641
Histological type	0.898199	0.736756	1.095018	0.288211				
T	1.075018	0.917442	1.259658	0.371066				
N	1.296917	1.125086	1.49499	0.000337	1.067364	0.815654	1.396751	0.634735
M	1.112185	0.727047	1.701341	0.623965				
Stage	1.229799	1.055993	1.432213	0.007796	0.919478	0.704143	1.200665	0.537464
Grade	1.280151	1.042481	1.572005	0.018423	1.185943	0.948725	1.482473	0.13421
HP infection	0.670859	0.475503	0.946476	0.023013	1.240031	0.818149	1.879458	0.310594
Reflux history	0.609943	0.439642	0.846213	0.00308	0.810763	0.567863	1.157561	0.248239
Residual tumor	1.482089	1.143333	1.921214	0.002962	0.992409	0.732518	1.344507	0.960774
Ethnicity	0.973056	0.672818	1.407272	0.884638				
Race	1.094327	0.885531	1.352353	0.403998				
Number of lymphnodes positive	1.058645	1.03801	1.07969	1.39E-08	1.019857	0.985855	1.055031	0.25574
Cancer status	2.480676	2.008099	3.064469	3.59E-17	1.796603	1.384077	2.332083	1.07E-05

Age	0.999936	0.985464	1.01462	0.993097				
Anatomic site	0.956627	0.828779	1.104196	0.544645				
Barretts esophagus	0.960004	0.676377	1.362567	0.819296				
